# The functional evolution of collembolan Ubx on the regulation of abdominal appendage formation

**DOI:** 10.1007/s00427-024-00718-0

**Published:** 2024-07-09

**Authors:** Yan Liang, Yun-Xia Luan

**Affiliations:** 1 Key Laboratory of Insect Developmental and Evolutionary Biology, CAS Center for Excellence in Molecular Plant Sciences, Shanghai Institute of Plant Physiology and Ecology, Chinese Academy of Sciences, Shanghai 200032, China; 2 University of Chinese Academy of Sciences, Beijing 100049, China; 3 Wellcome Sanger Institute, Wellcome Genome Campus, Hinxton, Cambridge CB10 1SA, UK; 4 Guangdong Provincial Key Laboratory of Insect Developmental Biology and Applied Technology, Guangzhou Key Laboratory of Insect Development Regulation and Application Research, Institute of Insect Science and Technology, School of Life Sciences, South China Normal University, Guangzhou 510631, China

**Keywords:** Collembola, Arthropoda, Appendage formation, RNA-seq, Transcriptional regulation, Functional evolution

## Abstract

*Folsomia candida* is a tiny soil-living arthropod belonging to the Collembola, which is an outgroup to Insecta. It resembles insects as having a pair of antennae and three pairs of thorax legs, while it also possesses three abdominal appendages: a ventral tube located in the first abdominal segment (A1), a retinaculum in A3, and a furca in A4. Collembolan Ubx and AbdA specify abdominal appendages, but they are unable to repress appendage marker gene *Dll*. The genetic basis of collembolan appendage formation and the mechanisms by which Ubx and AbdA regulate *Dll* transcription and appendage development remains unknown. In this study, we analysed the developmental transcriptomes of *F. candida* and identified candidate appendage formation genes, including *Ubx* (*FcUbx*). The expression data revealed the dominance of *Dll* over *Ubx* during the embryonic 3.5 and 4.5 days, suggesting that Ubx is deficient in suppressing *Dll* at early appendage formation stages. Furthermore, via electrophoretic mobility shift assays and dual luciferase assays, we found that the binding and repression capacity of FcUbx on *Drosophila Dll* resembles those of the longest isoform of *Drosophila* Ubx (DmUbx_Ib), while the regulatory mechanism of the C-terminus of FcUbx on *Dll* repression is similar to that of the crustacean *Artemia franciscana* Ubx (AfUbx), demonstrating that the function of collembolan Ubx is intermediate between that of Insecta and Crustacea. In summary, our study provides novel insights into collembolan appendage formation and sheds light on the functional evolution of Ubx. Additionally, we propose a model that collembolan Ubx regulates abdominal segments in a context-specific manner.

## Introduction

Extant arthropods are traditionally classified into four major groups: chelicerates, myriapods, crustaceans, and hexapods (including proturans, collembolans, diplurans, and insects) (Budd & Telford 2009; Giribet et al. 2001; Hughes & Kaufman 2002b). Throughout their evolution, arthropods gradually reduce abdominal appendages, particularly insects, which entirely lack these appendages in their adult stages (Jockusch & Smith 2015; Matsuda 2017). The establishment of a body plan is regulated by a cascade of orchestrated transcription factors during embryogenesis, including maternal effect genes, pair-rule genes, gap genes, and Hox genes (Peel et al. 2005). Hox genes encode transcription factors characterized by a helix-turn-helix DNA-binding homeodomain and play pivotal roles in the identification and appendage formation of each segment along the body axis of arthropods (Angelini & Kaufman 2005a, 2005b; Budd & Telford 2009; Hughes & Kaufman 2002a). Typically, Hox genes are organized in a cluster on the chromosome and exhibit both spatial and temporal collinearity in their expression patterns (Durston et al. 2011). *Dll* (*Distal-less*) serves as a marker gene for the appendage primordium in a wide variety of animals (Panganiban et al. 1997); within some groups of pancrustaceans (including crustaceans and hexapods) (Browne & Patel 2000; Cohen et al. 1991; Jockusch et al. 2004), it acts as a downstream target of Ubx (*Ultrabithorax*) (Buffry et al. 2023; Cohen 1990; Galant & Carroll 2002; Gebelein et al. 2002; Palopoli & Patel 1998; Ronshaugen et al. 2002). In *Drosophila melanogaster*, the Ubx gene generates six isoforms (*Ib, Ia, IIb, IIa, IVb, IVa*) via alternative splicing events, with variation in the architecture of the linker region between the YPWM motif and homeodomain (HD domain) (Geyer et al. 2015; Passner et al. 1999; Reed et al. 2010). Specifically, the *Ib* isoform (DmUbx_Ib) has the longest linker region, and the *IVa* isoform (DmUbx_IVa) does not contain the linker. Functional assays demonstrated that the longest linker is indispensable for the suppression of *Dll* expression (Gebelein et al. 2002); in addition, the QAQA domains and poly-Ala stretch located at the C-terminus are also crucial for the repression function (Galant & Carroll 2002; Gebelein et al. 2002; Hughes & Kaufman 2002b; Ronshaugen et al. 2002). In general, these structures facilitate the ability of Ubx to bind to the enhancer of *Dll* (Dll304) and consequently suppress the expression of the *Dll* gene (Cohen et al. 1993; Gebelein et al. 2002). In contrast, in branchiopod crustacean *Artemia franciscana*, Ubx (AfUbx) does not have the capacity to repress *Dll* expression (Ronshaugen et al. 2002). This functional shift can be attributed to the presence of phosphorylation sites within the C-terminus of AfUbx, which impedes its ability to suppress limb development (Galant & Carroll 2002; Ronshaugen et al. 2002). To date, the evolutionary processes and the molecular mechanism that give rise to the loss of abdominal appendages in adult hexapods remain elusive.

Collembola (springtails) is a group of basal hexapods, whose phylogenetic position is intermediated between aquatic crustaceans and terrestrial insects (Gao et al. 2008; Luan et al. 2005; Timmermans et al. 2008). Collembolans bear three distinct types of abdominal appendages (Fountain and Hopkin 2005): the ventral tube (or collophore) in abdominal segment 1 (A1), which is involved in osmoregulation with the external environment; the retinaculum in A3, a structure for holding the springing organ furca in place; and the furca in A4, a robust jumping apparatus (Tully & Potapov 2015). A previous study demonstrated that the collembolan Ubx specifies A1 and AbdA determines A4, and they both regulate the morphological formation of A2 and A3 (Konopova & Akam 2014). Intriguingly, collembolan Ubx, AbdA, and *Dll* are concurrently expressed in the collembolan abdomen during the embryonic stage, indicating that collembolan Ubx and AbdA do not repress *Dll* expression in abdominal segments (Palopoli & Patel 1998). However, the molecular mechanisms of how collembolan Ubx and AbdA interact with *Dll* and thus regulate appendage formation are unclear.

To address this question, we conducted a computational analysis of the developmental transcriptomes of Collembola, *F. candida* (Denmark strain), covering embryos and juveniles to adults. Through this analysis, we identified the genes potentially involved in appendage formation during embryogenesis, with *Ubx* being a notable inclusion. The expression data indicated that *Ubx* could not suppress *Dll* in the early appendage formation stages. Furthermore, we investigated the transcriptional regulatory functions of Ubx. Our findings revealed that the binding capacity and repression activity of *F. candida* Ubx (FcUbx) on *D. melanogaster Dll* are analogous to those of *D. melanogaster* Ubx (DmUbx_Ib) (Galant & Carroll 2002; Gebelein et al. 2002; Ronshaugen et al. 2002), and the regulatory mode and site(s) in the C-terminus of FcUbx resemble those of Ubx in the branchiopod crustacean *A. franciscana* (AfUbx) (Galant & Carroll 2002; Ronshaugen et al. 2002). Based on these data, we depicted a molecular functional evolutionary model of Ubx from crustaceans to basal hexapods to insects in the broad scope of panarthropods and proposed that the collembolan Ubx might exert its repression function in distinct abdominal segments in a context-specific manner.

## Materials and methods

### Sample collection and transcriptome sequencing

First, *F. candida* (Denmark strain) was synchronized for three generations: Some adult individuals (F_0_ generation) were transferred to a new Petri dish (10 cm), a container with a layer of a mixture of plaster of Paris and activated charcoal at a ratio of 9:1 by weight (Krogh 2009). After 24 h of oviposition, the eggs (F_1_ generation) were subsequently transferred to a new Petri dish for one month of culture to reach sexual maturity. The procedure was repeated twice to obtain synchronized adults of the F_3_ generation. Approximately 300 adults of the F_3_ generation were then transferred to three new Petri dishes for 24 h of oviposition, and all eggs were transferred to a new culture dish. Next, all the eggs laid by the F_3_ generation were collected and transferred to new culture dishes (5 cm) every 24 h, except on the last day, when eggs were collected at 12 h after the adults had been transferred.

After 51 days of collection and culture, 51 samples were obtained, ranging from day 0.5 to day 50.5. Fifteen developmental samples, including eggs (from days 0.5 to 9.5), juveniles (days 12.5, 19.5 and 28.5), and adults (days 31.5 and 45.5), were chosen for transcriptome sequencing. RNA was prepared and extracted separately from 15 samples by using a miRNeasy mini kit (QIAGEN, Germany) according to the manufacturer’s instructions. The transcriptomes were sequenced on the BGISEQ-500 platform by BGI (Yuzuki 2015) (Shenzhen, China). All of the raw reads had been published (BioProject PRJNA433725) (Liang et al. 2019).

### Transcriptome analysis and gene annotation

The reads mapping and differential gene expression analysis methods used were identical to the previous study (Liang et al. 2019). The genome we used was non-chromosome-level genomic data from the published *F. candida* genome (Luan et al. 2023). The genome index was built using Bowtie2 (Langmead & Salzberg 2012), and the reads were mapped using tophat2 (Kim et al. 2013) The transcript abundance (RPKM) was estimated using cufflinks (Trapnell et al. 2012), with the gene annotation file from the whole genome. Gene Ontology (GO) annotations were accomplished by running BLAST2GO (Conesa et al. 2005) against the UniProt database (UniProt release 2018_02) (Bairoch et al. 2005). The expression profile was obtained from the data matrix representing the expression abundance (RPKM, reads per kilobase of transcript per million fragments mapped) from 15 transcriptomes. The distribution of gene expression was visualized as Ridgeline Plots in “ggplot2” (Wickham 2016). Additionally, to explore the relationships among developmental stages, we employed several analyses. Principal component analysis was conducted using the “prcomp” function; Pearson correlation analysis was executed using the “cor” function in R. For hierarchical clustering analysis, we first scaled the dataset using the “Euclidean” method and then applied the “hclust” function with the “ward.D2” method. Data visualization was achieved using “ggplot2” and “pheatmap” (Kolde 2012) in RStudio (2023.3.0.386) (RStudio 2020).

### Mining of genes involved in appendage formation

To identify genes associated with appendages formation from time-series bulk RNA-seq data, we employed the Short Time-series Expression Miner (STEM) approach (Ernst & Bar-Joseph 2006; Ernst et al. 2005), which attempts to assign genes to a previously defined temporal trajectory/development (Bar-Joseph et al. 2012). Through the embryonic observation, PCA analysis, and hierarchical clustering, we selected four key time points: day 1.5 (E_1.5d, blastula, no appendages), day 3.5 (E_3.5d, early stage of appendage formation), day 5.5 (E_5.5d, mid-stage of appendage formation), and day 7.5 (E_7.5d, mature stage of appendage formation) for mining genes were involved in appendage formation. STEM software was used to classify all the clusters according to the abundance of gene expression. Subsequently, genes in the clusters that were supposed to correlate with appendage formation were sorted out and then annotated by Blast2GO software (Conesa et al. 2005).

### Expression profiling of Hox genes and *Dll* in *D. melanogaster* and *F. candida*

The gene expression profile of *D. melanogaster* was downloaded from flybase (http://ftp.flybase.org/releases/current/precomputed_files/genes/gene_rpkm_matrix_fb_2023_05.tsv.gz) (Brown et al. 2014). The expression values of Hox genes and *Dll* were subset from the expression profile of *D. melanogaster* and *F. candida*. Notably, the *Ubx* in *F. candida* was fragmented into five transcripts (XLOC_011518, XLOC_011661, XLOC_011777, XLOC_011778 and XLOC_011779) (Supplementary Data 1). To calculate the expression of *Ubx*, we computed and accumulated the RPKM of those transcripts. The normalization of gene expression was performed via two approaches. In normalized RPKM, the original RPKM was normalized by comparing the minimum and maximum of each gene throughout the embryonic stages to elucidate the expression pattern and the lowest and highest expression stage of a gene. In Z-score, the original RPKM was normalized by the z-scale in R (RStudio 2020), which can reflect the expression pattern of all the selected genes.

### Mining of genes correlated with *Ubx* from transcriptomes

To predict the putative function or the underlying regulation of Ubx, based on the gene expression profile, we used the Spearman correlation algorithm (Spearman correlation coefficient r > 0.9, *p* < 0.01) to mine genes coexpressed with *Ubx* during embryogenesis. The coexpressed genes were extracted and then annotated by Blast2GO of gene ontology terms (GO terms) and KEGG pathway annotation (Conesa et al. 2005; Kanehisa & Goto 2000).

### Acquisition of *Ubx, Exd*, and *Hth* sequences

The complete sequences of two isoforms of *Ubx* in *F. candida* (Collembola) and the partial sequences from *Sinentomon erythranum* (Protura) and *Campodea augens* (Diplura) were cloned by degenerate PCR from the homeodomain, further obtained by 5′ RACE and 3′ RACE. Specifically, two isoforms of FcUbx were validated by using exon–intron and exon-exon junction PCR with overlapping primers spanning the linker region. *Exd* and *Hth* were identified via BLAST (Altschul et al. 1990; McGinnis & Madden 2004) from transcriptomes and further validated by PCR cloning. All sequences were submitted to NCBI database (OR593736, OR593737, OR593738, OR593739, OR604006, OR604007). The sequences of Panarthropod Ubx were aligned by MAFFT (Rozewicki et al. 2019), and the gaps were removed manually. The multiple sequence alignment was visualized in Jalview (Waterhouse et al. 2009).

### Protein expression and purification

The N-terminus truncated collembolan proteins, FcU1, FcU2, Exd, and Hth, used for protein expression were constructed in the pGEX-4t-1 plasmid (with the GST-tag inserted) and transformed into *Escherichia coli* competent cells (the BL21 strain). Protein expression was first started from 500 ml of Luria–Bertani (LB) medium (pH = 7) cultured at 37 °C at 220 rpm. Until it reached the exponential growth phase (OD600 = 0.8 ~ 1.0), isopropyl β-D-1-thiogalactopyranoside (IPTG) was added (0.1 mM) to 2 l of LB medium. The whole culture was then subjected to low-temperature induction of protein expression at 16 °C and 220 rpm for 12 h. All the GST-tagged proteins were purified under native conditions according to the manufacturer’s instructions (Sangon Biotech, Shanghai, China). All proteins were quantitated by comparison to a set of bovine serum albumin (BSA) concentration gradients: 750, 500, 250, 125, 100, 75, 50, 25, and 0 ng/μl by Coomassie blue staining and further confirmed by anti-GST western blotting.

### Electrophoretic mobility shift assays (EMSAs)

We identified a putative FcDll element (PFE) in *F. candida* through a search within the approximately 4000 bp intergenic genomic region upstream of the first exon of the collembolan *Dll* gene. This search utilized the Hox binding consensus sequence (5′-TATA-3′) (Berger et al. 2008; Ekker et al. 1991; Noyes et al. 2008) and the Exd binding consensus sequence (5′-TGAT-3′) (Ryoo et al. 1999; Slattery et al. 2011).

For EMSAs, the DMXR element (Gebelein et al. 2002, 2004), a repression regulatory element of *Dll* (Dll304) in *D. melanogaster*, and the putative regulatory element of *Dll* in *F. candida* (putative FcDll element, PFE) were utilized as a DNA probe in each assay separately. The probe was first synthesized as two separate primers (forward and reverse strands), which were tagged with Cy5 at the 5′ end. The primers were diluted to a concentration of 1 μM. Subsequently, 50 μl of each primer was mixed and incubated at 95 °C and then cooled to room temperature. The primers were annealed, resulting in the formation of double-stranded DNA probes. Twenty nanograms of DNA probe was used in each EMSA, and the amount of protein used in each EMSA was 10 pmol of GST; 0.2, 1, and 1.5 pmol of FcU1; 0.4, 2, and 3 pmol of FcU2; and 1 pmol of Exd and Hth. The procedures for electrophoretic mobility shift assays (EMSAs) were adjusted and visualized according to the protocol (He et al. 2016). The 20-μl EMSA solution consisted of 5 × EMSA buffer (Tris (0.1 M), glycerol (25%), and BSA (0.2 mg/ml)), 2.5 M MgCl_2_, 1 M DTT, double-distilled water, poly(dI-dC), protein, and Cy5-labelled DNA probe. The reaction was incubated for 25 min at 25 °C. The 12% non-denaturing polyacrylamide gel contained double-distilled water, 5×TBE buffer, 50% glycerol, polyacrylamide (the monomer: dimer ratio was 80:1), 10% ammonium persulfate, and tetramethyl ethylenediamine (TEMED). Electrophoresis was carried out on ice, with the blank gel pre-electrophoresed at 120 V for 1 h. Subsequently, 20 μl of the reaction mixture was loaded into each vial of the gel, and the samples were run at 150 V for 1–1.5 h. For reaction visualization, the entire gel with the glass container was scanned directly with the A Starion FLA-900 phosphorimager (Fujifilm, Japan).

### *Drosophila* S2 cell transfection and dual luciferase reporter assays

The complete sequences of *D. melanogaster* Ubx (DmUbx_Ib, DmUbx_IVa) and collembolan Ubx (FcU1, FcU2) and the truncated collembolan Ubx (FcU1/△C, FcU2/△C) and chimeric Ubx of *Drosophila* and collembolan (Dm/Fc_L, Dm/Fc_C) were cloned and inserted into the pAC5.1 expression vector. To test the repression on DMXR, this regulatory sequence was constructed into the pGL3-promoter vector (Promega), which contains the SV40 enhancer and firefly luciferase. Transfection experiments were carried out using the Qiagen Effectene reagent (Qiagen, Germany), according to the manufacturer’s protocol. Initially, *Drosophila* S2 cells were cultured in Schneider’s *Drosophila* medium (Sigma-Aldrich, USA) supplemented with 10% foetal bovine serum (HyClone, USA). After 24 h, the cells were aliquoted into 48-well plates at 150 μl per well. After 12 h, 50 μl of DNA-enhancer mixture, containing 1.2 μl of enhancer (the reagent from the Effectene reagent kit), 0.075 μg of pGL3-promoter vector, 0.075 μg of pAC5.1 vector, and 0.2 μl of the Renilla fluorescent vector (pRL), was added to the mixture, followed by an 8-min incubation. Then, 4 μl of Effectene and 200 μl of cell culture medium were added, and the transfected cells were cultured for 48 h in a 27 °C incubator. Each protein vector was set with technical triplicates.

The dual luciferase reporter assay kit (Promega, USA) was used to measure the reporter gene expression, and the luciferase activities were detected by a Modulus™ Micro-plate Luminometer (Turner BioSystems, USA). The repression activity of each protein is represented by the average ratio of Firefly:Renilla luciferase activity. To estimate the relative repression of the proteins on the DMXR, we compared the repression activity of each sample with that of DmUbx_Ib. For pairwise comparisons, we performed Student’s *t*-test in R (RStudio 2020).

## Results

### Development and time-course transcriptomes of *F. candida*

**Fig. 1:**
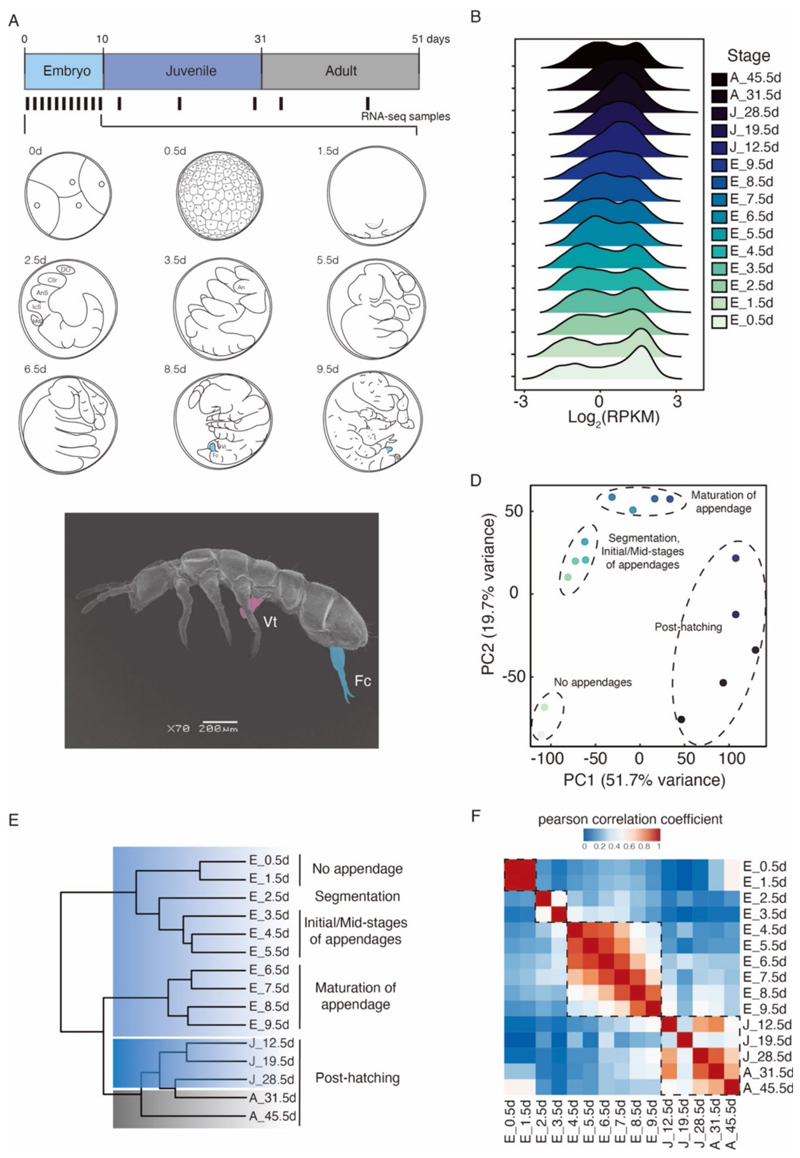
Analysis of developmental transcriptomes of *F. candida*. **A** Schematic of samples used for developmental transcriptome sequencing and the observation of embryonic development. Whole-animal collections were made from embryos (blue), juveniles (grey blue) and adults (grey). The bars indicate that different developmental samples were collected. 0d (d, days after oviposition): four-cell stage; 0.5d: blastula stage; 1.5d: gastrula stage; 2.5d: Initial phase of tissue differentiation stage; 3.5–6.5d: middle phase of tissue differentiation stage; 7.5–8.5d: late phase of tissue differentiation stage or maturation of appendage; 9.5d: prehatching stage. An, antenna; AnS, antenna segment; Cllr, clypeolabrum; DO, dorsal organ; Fc, furca (in blue colour); IcS, intercalary segment; MdS, mandibular segment; Vt, ventral tube (in pink colour). **B** Distribution of gene expression of 25,038 genes from 15 developmental transcriptomes. RPKM, reads per kilobase per million, normalizes the raw count by transcript length and sequencing depth. **C** Scanning electron microscopy (SEM) image of a whole animal of adult *F. candida*. Vt, ventral tube (shade in pink colour); Fc, furca (shade in blue colour). **D** Principal component analysis of the developmental transcriptomes. The dashed ellipses indicate the stages of appendage development. **E** Simplified hierarchical clustering of all developmental transcriptomes. The stages of appendage development are shown on the right. **F** Heatmap of the Pearson correlation coefficient of all the samples; the dashed rectangles indicate the relationship of samples

The embryos of *F. candida* (Denmark strain) took approximately 10 days to reach the juvenile stage when incubated at 21 °C (Fountain and Hopkin 2005)). Referring to the observation of the embryonic developmental stages in *F. candida* (Shanghai strain) (Gao et al. 2006), the samples selected for sequencing are illustrated in Fig. 1A. The embryos at the 0–0.5 day (E_0.5d) predominantly ranged from the four-cell stage to the blastula stage. By 1.5 days, most embryos had progressed to the gastrula stage. In 2.5 days, the initial phase of tissue differentiation stage was characterized by the segmentation and formation of appendage primordia. At 3.5 days, the antenna and thorax appendages started segmentation and elongation; the furca was observed by 4 days (Gao et al. 2006). During the period of 4.5 to 6.5 days, the middle phase of the tissue development stage progressed, accompanied by the growth and maturation of appendages. From 7.5 to 8.5 days, in the late phase of tissue differentiation, the appendages were fully formed, and the animals were preparing for hatching. Finally, at 9.5 days, the animals were actively moving within the eggshell, and some individuals had already hatched. Typically, it took these juveniles 1 month to reach sexual maturity as adults (Fig. 1A, C).

The analysis of developmental transcriptomes recaptured the expression of a total of 25,803 genes throughout the developmental process (Fig. 1B; Supplementary Data 1). Transcriptomic analyses elucidated that the relationships among all the developmental samples were consistent with the observation of development (Fig. 1A, D, E, F). In general, all the post-hatching samples (juveniles and adults) clustered together, demonstrating that the hatching event acted as a critical developmental transitional event (Fig. 1D, E, F). The embryonic samples were grouped separately: In the early stages E_0.5d and E_1.5d, no appendages were observed, and these two stages were grouped; the embryonic stages E_2.5d and E_3.5d, particularly marked by the segmentation and formation of appendage primordia, were clustered, and the period spanning from E_4.5d to E_9.5d, deemed the development of appendages, formed a distinct group. Specifically, E_4.5d to E_5.5d represent the mid-phase of appendage formation, and E_6.5d to E_9.5d were identified as the maturation of appendage formation (Fig. 1D, E, F).

### Mining of genes related to appendage development in *Collembola*

Springtails bear three pairs of thoracic legs and appendages in the abdomen (Fig. 1C). These abdominal appendages, however, exhibit distinct morphological characteristics from each other (Fig. 1C). To identify genes associated with the establishment of appendages, especially the abdominal appendages, we conducted a Short Time-series Expression Miner analysis (STEM analysis) (Ernst & Bar-Joseph 2006), which would assign genes to one of several previously defined developmental trajectories (Bar-Joseph et al. 2012). Based on the embryonic observation (Fig. 1A) and transcriptomic analyses (Fig. 1D, E, F), we deemed the formation of appendages as four stages for our analysis: E_1.5d, corresponding to the blastula stage without any appendage structure; E_3.5d, representing the early stage of appendage development, marked by the emergence of the appendage primordia; E_5.5d, indicating the middle stage of appendage development; and E_7.5d, the late stage of appendage development.

**Fig. 2:**
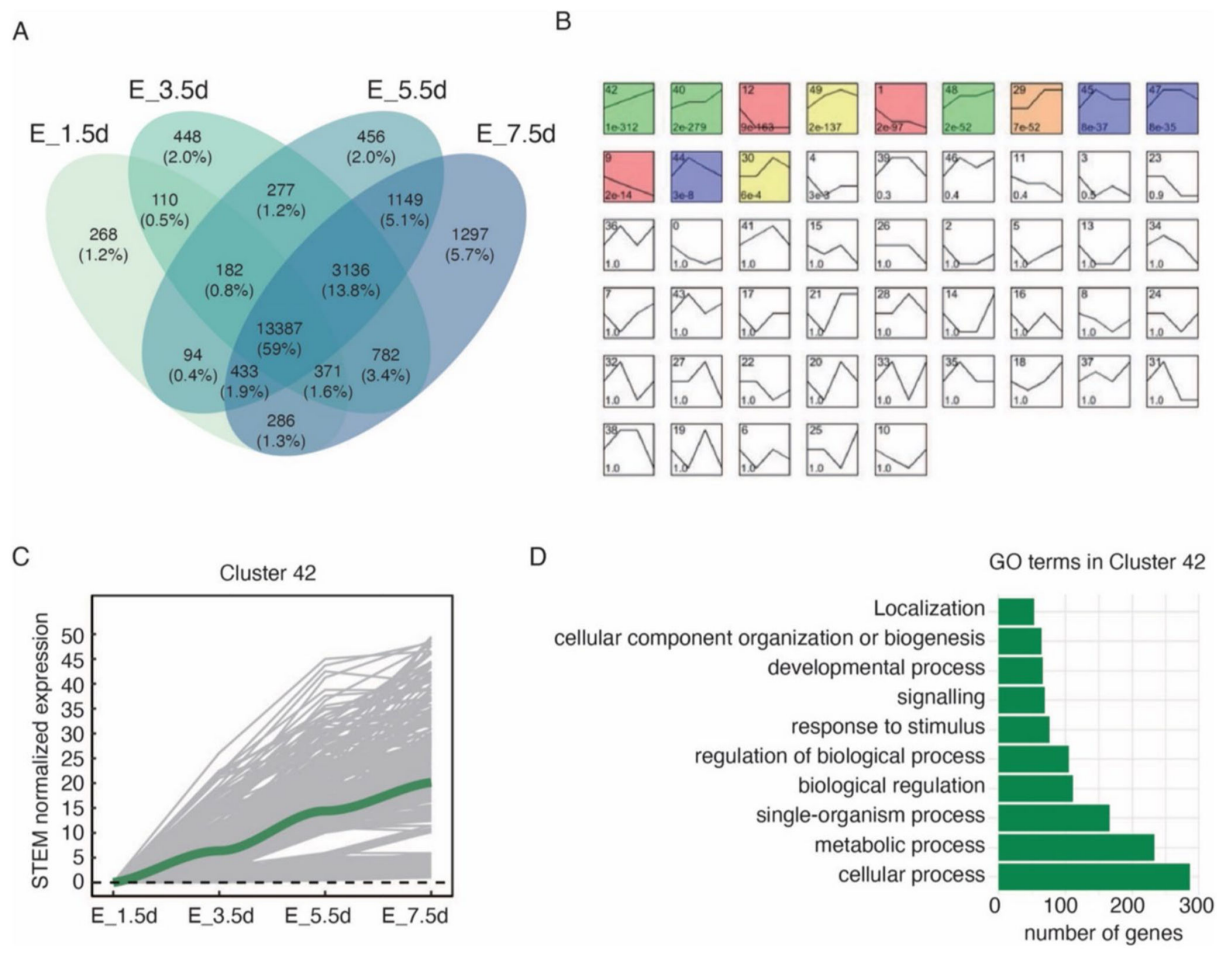
Identification of genes involved in appendage formation. **A** Venn diagram illustrating the number and percentage of expressed genes of each intersection among the four appendage formation stages. **B** A screenshot of different gene expression profiles identified by Short Time-series Expression Miner (STEM) analysis. The number of each profile is shown at the upper left in the square. The coloured profiles indicate that genes were clustered significantly (permutation test, *p* < 0.001). **C** The STEM normalized expression of cluster 42. Values higher than 50 were excluded. **D** Annotated GO terms of cluster 42

The number of expressed genes (RPKM > 0) in these four stages was 15,131, 18,693, 19,114 and 20,841 genes, respectively (Fig. 2A; Supplementary Data 1, 2). The STEM analysis automatically categorized these genes into 50 profiles, among which 12 clusters were significantly statistically enriched (permutation test, *p* < 0.001). According to the morphological observations (Fig. 1A) and the developmental trend we defined, the emergence of appendages was characterized by gradual development, with the transition of gene expression levels from low to high (Fig. 2B). Cluster 42, which reflected this trend, was selected for further investigation (Fig. 2C). Cluster 42 contained a total of 865 genes and 2,318 transcripts, of which 1229 were annotated by Blast2GO (Supplementary Data 3). These genes were annotated as 10 biological processes of GO terms (Fig. 2D) and involved in 32 KEGG pathways (Supplementary Data 3). In particular, 36 transcripts were annotated in the developmental process, and 18 genes are the morphogenesis and appendage-related genes (Table 1; Supplementary Data 3). These genes constitute a set of conventional hierarchical developmental genes (Peel et al. 2005), including the body patterning gene *Notch* (de Celis et al. 1998; Kurata et al. 2000; O’Day 2006; Rauskolb & Irvine 1999) and Engrailed (Brower 1986; van de Heuvel et al. 1993); the limb proximal–distal axis formation genes *Exd* and *Hth* (Wu & Cohen 1999); and the body plan “selector genes”, the Hox genes *Ubx, Antp*, and *Scr* (Weatherbee & Carroll 1999). This cluster also comprised genes relevant to muscle and actin function, for instance, *MYO1E* (*Myo61F*, orthologous in *D. melanogaster*) which is an unconventional myosin-like protein (McIntosh & Ostap 2016); *Lhx1* (*Lim1*, orthologous in *D. melanogaster*), which regulates tarsal segments (Natori et al. 2012); *FBN2* (*CG7526*, orthologous in *D. melanogaster*) which involves in fibrosis (Mead et al. 2022).

In summary, STEM analysis effectively identified several genes closely associated with appendage formation, thus validating the predefined stages used for identifying appendage formation genes and providing supportive evidence for our approach.

### Dominance of *Dll* over *Ubx* during appendage formation in *F. candida*

**Fig. 3:**
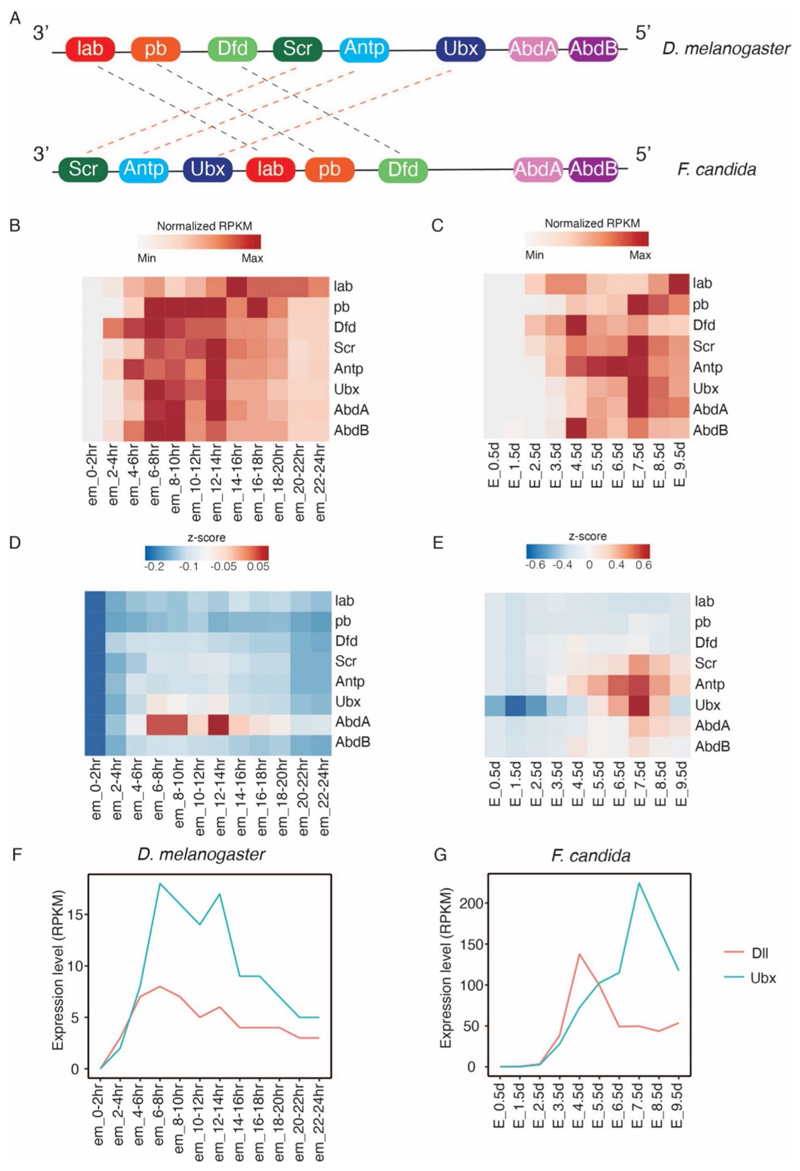
Comparison of the Hox cluster and *Dll* gene expression between *D. melanogaster* and *F. candida*. **A** Genomic architecture of the Hox cluster in *D. melanogaster* (refined from Pearson, et al. 2005) and *F. candida* (refined from Faddeeva-Vakhrusheva, et al. 2017). The distances between genes were not scaled in proportion to the original genomic distance. The dashed lines indicate that the Hox genes are rearranged. **B**–**G** Expression profiles of Hox genes and *Dll* in *D. melanogaster* (**B, D, F**) and *F. candida* (**C, E, G**). Normalized RPKM, the RPKM were normalized by the minimum and maximum expression value of each gene throughout embryonic stages. Z-score, the RPKM values were normalized by z-scale

In general, the Hox cluster is characterized by spatial (Fig. 3A) and temporal collinearity (Monteiro & Ferrier 2006). However, previously reported genome assemblies of *F. candida* (Faddeeva-Vakhrusheva et al. 2017; Luan et al. 2023) reveal that *Scr, Antp*, and *Ubx* inserted into the anterior region of the Hox complex, indicating a lack of spatial collinearity (Fig. 3A) (Faddeeva-Vakhrusheva et al. 2017). Our expression analysis of Hox genes in *D. melanogaster* also reveals a lack of temporal collinearity (Fig. 3B), consistent with the founding of previous study (Gaunt 2015). Similarly, the Hox genes in *F. candida* did not display temporal collinearity (Fig. 3D). For instance, the *pb* gene was transcribed at E_3.5d, occurring later than *Dfd* and *Scr*, and the most posterior gene *AbdB* was highly expressed at the early stage of E_4.5d. Notably, *Ubx* specifically displayed the highest expression levels at the appendage maturation stage E_7.5d.

In *D. melanogaster*, Ubx acts as a *Dll* repressor during embryogenesis in the abdominal segments (Gebelein et al. 2002). The transcriptomic expression data demonstrated that Drosophila *Ubx* exhibited higher expression levels than *Dll* (Fig. 3F) during embryogenesis, suggesting its role in repressing *Dll*. Conversely, in *F. candida, Dll* predominated over *Ubx* during the E_3.5d and E_4.5d stages (Fig. 3G), indicating that FcUbx may not repress *Dll* during the appendage formation stage. However, from the E_5.5d stage onward, *Ubx* expression surpassed that of *Dll*, indicating the potential of Ubx to regulate or suppress *Dll* during the appendage maturation.

### Collembolan Ubx can bind to Hox/Exd/Hth DNA binding motifs

Ubx regulates *Dll* through DNA binding and transcriptional repression (Galant & Carroll 2002; Gebelein et al. 2002; Grenier & Carroll 2000; Ronshaugen et al. 2002). However, the mechanism through which the collembolan Ubx interacts with *Dll* has not been determined. To address this, we first obtained two complete isoforms of *Ubx* from *F. candida*, which are produced by alternative splicing and vary at the linker region (FcUbx, isoform 1, FcU1 with a linker of GQSYL; isoform 2, FcU2 without a linker) (Supplementary Data 4), and investigated their binding capacity and repression activity through in vitro and in vivo assays.

**Fig. 4:**
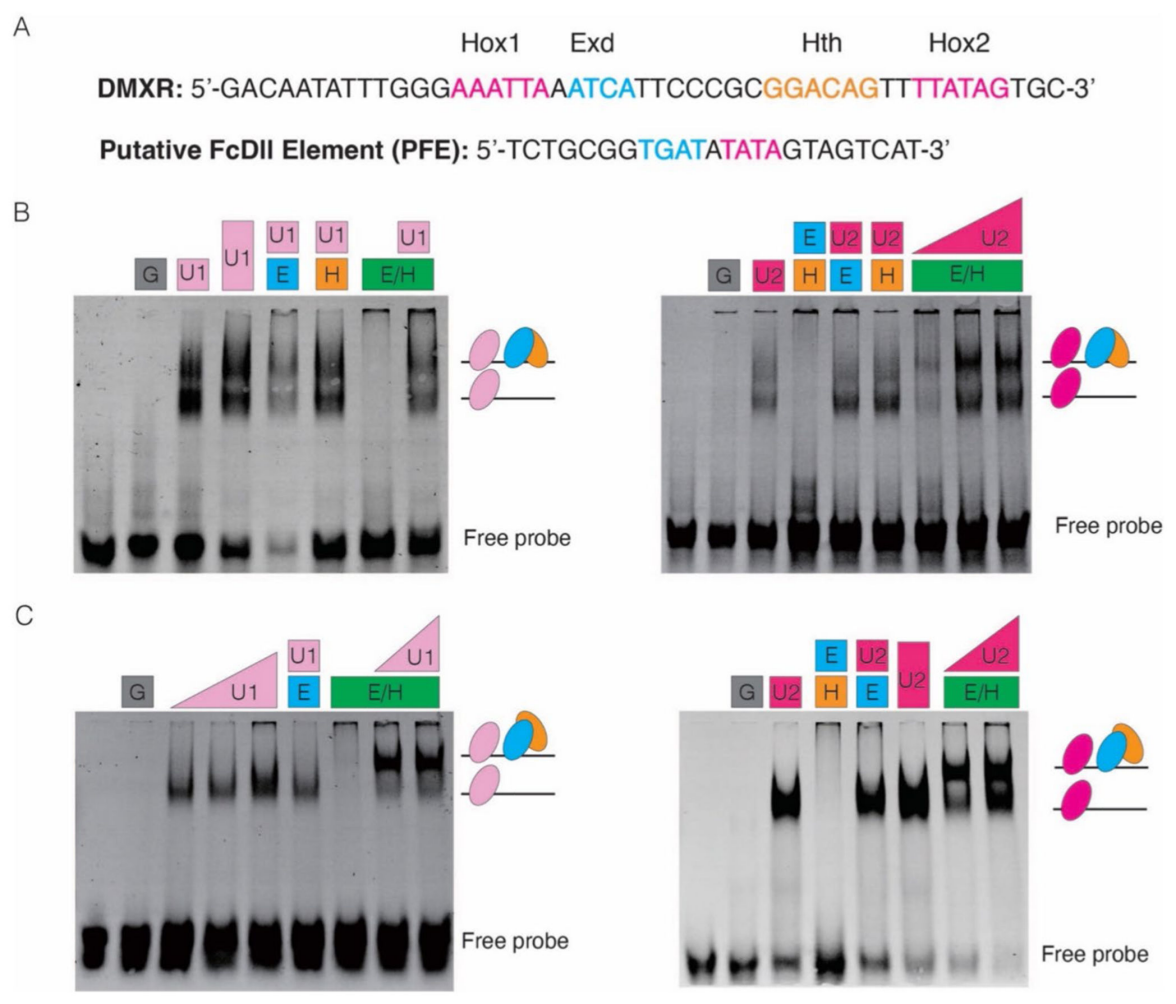
The collembolan Ubx and its cofactors could bind to the DNA elements that contain Hox/Exd/Hth binding motifs. **A** DNA probes used for EMSAs. DMXR, the transcription regulatory element of *D. melanogaster Dll* (Gebelein et al. 2002, 2004); putative FcDll element (PFE), a screened DNA region of *F. candida*, containing the Hox and Exd binding motifs. The binding sites of Hox/Exd/Hth are shown in colours. **B, C** Assemblies of collembolan Ubx/Exd/Hth on DMXR and putative FcDll element (PFE), respectively. G, GST; U1, FcU1; U2, FcU2; E, Exd; H, Hth. Simplified complexes are indicated on the right

We conducted the electrical mobile shift assays (EMSAs) to examine the binding capacity of the collembolan Ubx on an exogenous *Dll* element, the *Dll* regulatory element of *D. melanogaster* (DMXR, the repression element on Dll304) (Gebelein et al. 2004) (Fig. 4A). The proteins of two isoforms of collembolan Ubx (FcUbx1, FcUbx2) can readily bind to DMXR, demonstrating that FcUbxs exhibit binding capability and that their linker region does not affect this binding ability (Fig. 4B). Furthermore, the dimer Exd/Hth stimulates Ubx binding to DNA and forms Ubx/Exd/Hth trimeric protein complexes (Fig. 4B), thereby demonstrating the ability of collembolan Ubx/Exd/Hth complexes to bind exogenous *Dll* in vitro.

To test the binding capacity of the collembolan Ubx/Exd/Hth complex on its *Dll* DNA, we searched for *Dll* regulatory elements within approximately 4000 bp of the intergenic genomic region upstream from the first exon of *Dll* (Supplementary Data 6). We identified a binding motif containing both Hox/Exd binding sites and referred to this element as the putative FcDll element (PFE) (Fig. 4A; Supplementary Data 6). The EMSAs show that FcUbx1 and FcUbx2 cooperatively bind with Exd and Hth on this DNA element (Fig. 4C), providing additional evidence that collembolan Ubx is proficient in binding DNA defined as the endogenous putative Hox/Exd binding element. Nonetheless, owing to the absence of practical genetics tools for collembolans, the validation of whether this DNA element serves as the regulatory region of *Dll* in collembolans remains unattainable.

We conclude that the collembolan regulatory trimeric complex Ubx/Exd/Hth can effectively bind to the Hox/Exd/Hth DNA binding motifs, and that the lack of *Dll* repression of collembolan Ubx does not appear to be related to any deficiencies in DNA binding capacity. This finding implies that the mechanisms underlying the absence of repression may stem from other aspects of the regulatory process.

### The C-terminus of FcUbx contains both repression and regulatory domains

**Fig. 5:**
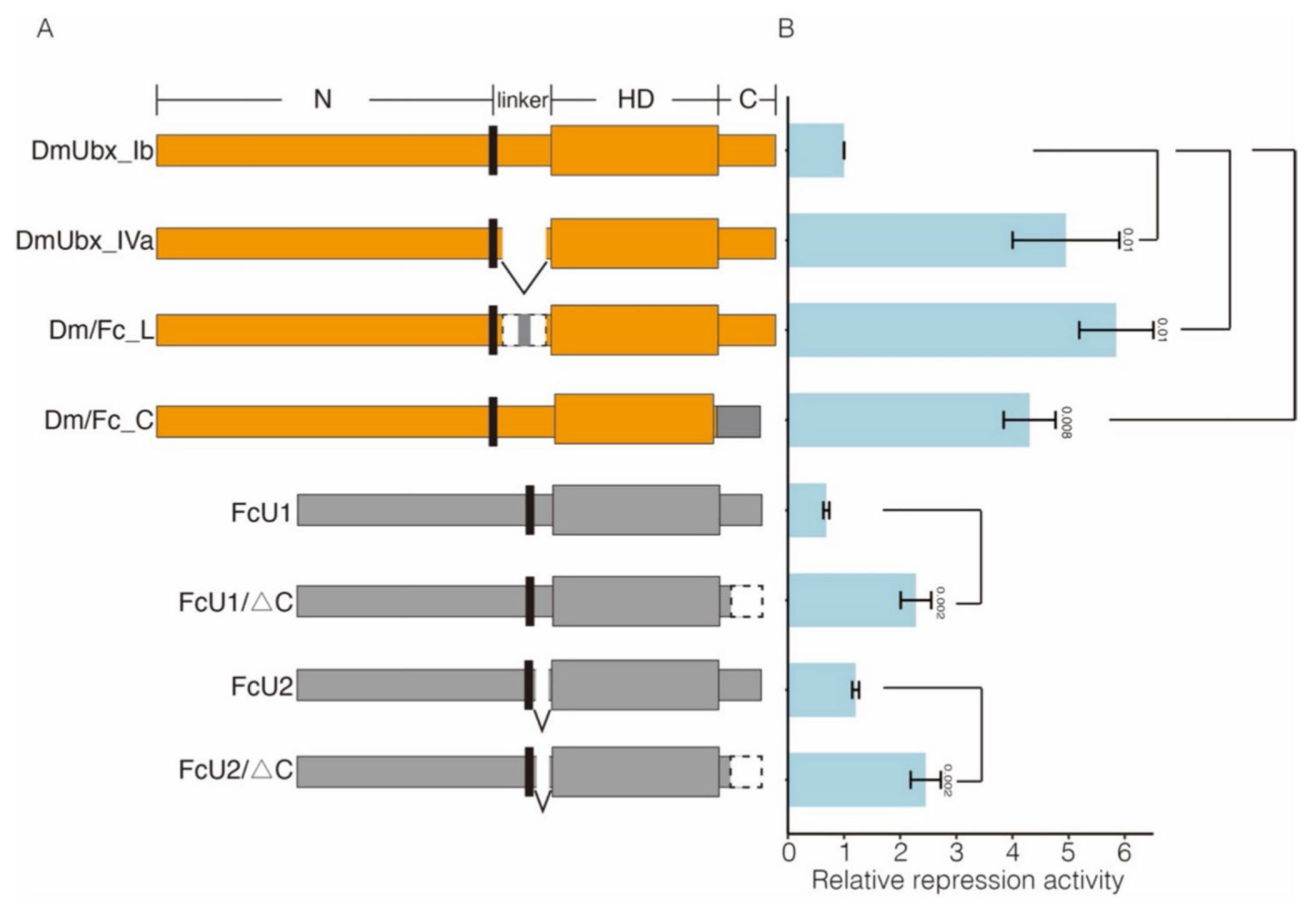
The collembolan Ubx could repress *Dll* transcription while its C-terminus contains regulatory domain(s). **A** Constructs of proteins used for transcription repression assays, including complete sequences, chimaeras, and truncations of *D. melanogaster* (yellow shade) and *F. candida* (grey shade) Ubx. Dm/Fc_L, the linker of DmUbx_Ib was replaced by the linker (GQSYL) of FcU; Dm/Fc_C, the C-terminus of DmUbx_Ib was replaced by the C-terminus (AKADCKSVY) of FcU; FcU1△C and FcU2△C, the C-terminus (QAQA AKADCKSVY) of FcUbx was deleted. **B** The relative transcriptional repressive activity of Ubx. The number on the *Y*-axis indicates transcriptional repression activity relative to that of DmUbx_Ib. Significant *p* values of selected pairwise comparisons are shown (Student’s *t*-test)

Next, to evaluate the transcriptional activity of the collembolan Ubx on *Dll*, we carried out dual luciferase assays in *Drosophila* S2 cells. This involved assessing the repression capacity of various proteins (Fig. 5A; Supplementary Data 5), including the complete, truncated, and chimeric forms of both collembolan and *Drosophila* Ubx proteins on the expression of the firefly luciferase reporter gene under the regulation of the DMXR element (Figs. 4A and 5A; Supplementary Data 7).

Although previous research has shown that collembolan Ubx cannot repress *Dll* (Palopoli & Patel 1998), our results surprisingly revealed that the two isoforms of the collembolan Ubx (FcU1 and FcU2) were capable of inhibiting the expression of the firefly luciferase reporter gene (Fig. 5B), which could support our hypothesis that Ubx could repress *Dll* expression during the stages of appendage maturation (Fig. 3G). For collembolan Ubx, the linker region is not required for the repression function; In contrast, the longest linker is indispensable for the repression function of *Drosophila* Ubx, as the non-linker isoform of *Drosophila* Ubx (DmUbx_IVa) and the chimeric protein Dm/Fc_L (linker of *Drosophila* Ubx is replaced by a short linker GQSYL from *F. candida*) significantly lost its repression capability (Fig. 5B).

**Fig. 6:**
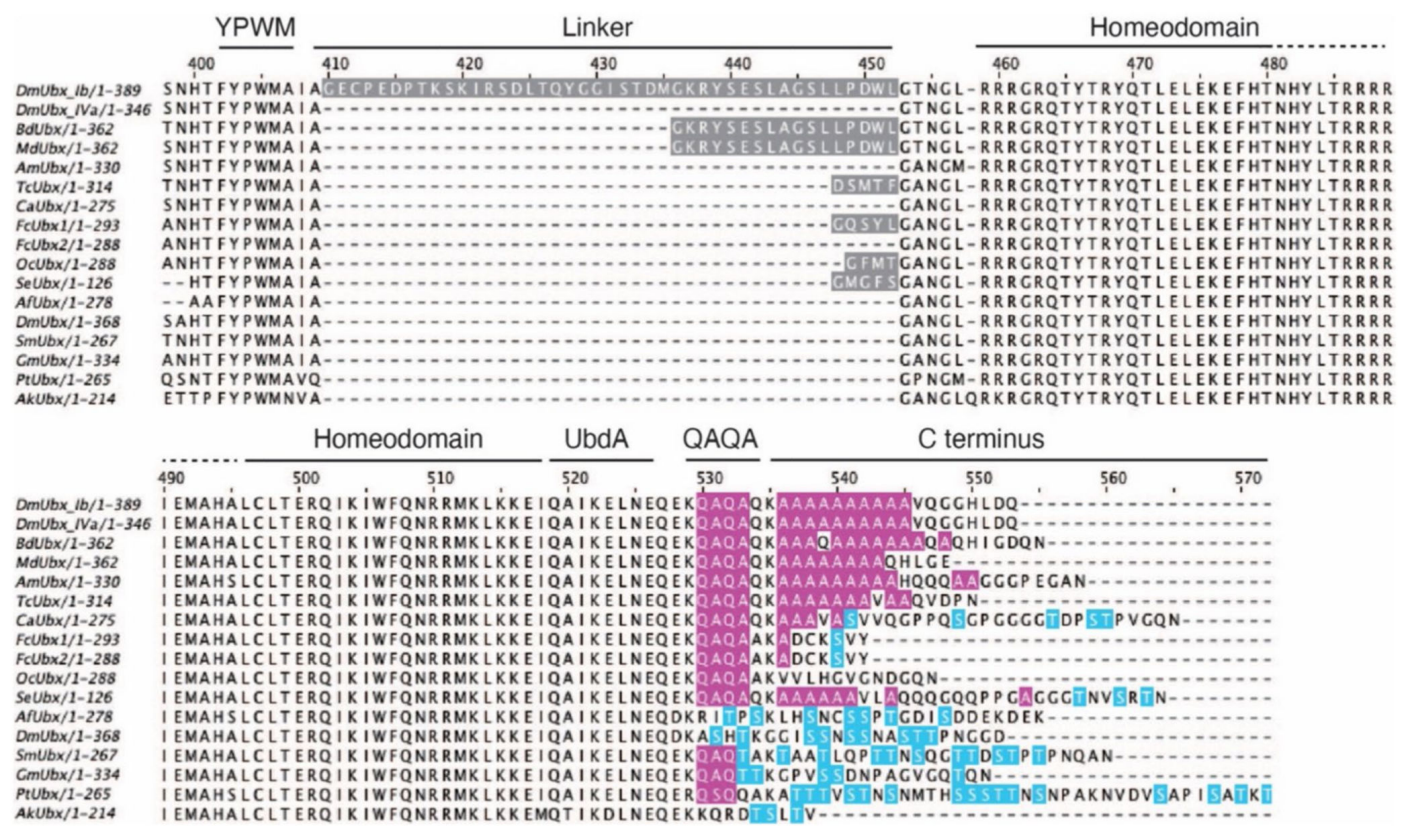
Sequence comparison of the linker and C-terminus of the panarthropod Ubx. Partial multiple sequence alignment of Ubx across Panarthropoda, encompassing Insecta, Diplura, Collembola, Protura, Crustacea, Myriapoda, Chelicerata and Onychophora. Insecta: *Drosophila melanogaster* (DmUbx_Ib, DmUbx_IVa), *Bactrocera dorsalis* (BdUbx), *Musca domestica* (MdUbx), *Apis mellifera* (AmUbx), *Tribolium castaneum* (TcUbx); Diplura: *Campodea augens* (CaUbx); Collembola: *Folsomia candida* (FcUbx1, FcUbx2), *Orchesella cincta* (OcUbx); Protura: *Sinentomon erythranum* (SeUbx); Crustacea: *Artemia franciscana* (AfUbx), *Daphnia magna* (DmUbx); Myriapoda: *Strigamia maritima* (SmUbx), *Glomeris marginata* (GmUbx); Chelicerata: *Parasteatoda tepidariorum* (PtUbx); Onychophora: *Acanthokara kaputensis* (AkUbx). The functional domains are indicated. YPWM, YPWM motif; UbdA, UbdA motif. Features of sequences are highlighted in colours. The QAQA domain and poly-Ala stretch are marked in pink, and putative phosphorylated sites S/T (Ser/Thr) are marked in blue. Linkers are marked in grey

Gebelein et al. revealed that the truncated form of the *Drosophila* Ubx C-terminus is partially able to repress *Dll* (Gebelein et al. 2002). In contrast, our repression assay showed that the truncation of FcU1 and FcU2, which lack a C-terminus (FcU1△C and FcU2△C), significantly decreased their repression ability (Fig. 5B), indicating that the C-terminus may contain a potential transcriptional inhibitory domain. Unexpectedly, the chimeric protein Dm/Fc_C (the C-terminus of *Drosophila* Ubx is replaced by a short C-terminus of AKADCKSVY from *F. candida*) exhibited a substantial loss of repression capacity when compared that of DmUbx_Ib, suggesting that the C-terminus of collembolan Ubx (AKADCKSVY) probably contains putative regulatory or modification site(s) that are capable of regulating the repression function of DmUbx_Ib (Figs. 5 and 6B).

Collectively, our results indicate that the linker of FcUbx is unnecessary for the repression function, while the C-terminus may contain both a repression domain (QAQA domain) and a functional regulatory site (S) (Fig. 6). These findings provide functional evidence for the mechanisms of Ubx-mediated gene repression.

### The sequence evolution of linker and C-terminus in arthropod Ubx

The regulatory functions of the linker and C-terminus of Ubx in *D. melanogaster* and *F. candida* suggest their significant roles in the evolution and regulation of arthropod abdominal appendage formation. To depict the evolutionary trajectory of those sequence features, we compared Ubx sequences from diverse representatives of panarthropods, including Insecta, Diplura, Collembola, Protura, Crustacea, Myriapoda, Chelicerate, and Onychophoran (Supplementary Data 4).

As illustrated in the multiple sequence alignment of Ubx across panarthropods (Fig. 6), *D. melanogaster* (DmUbx_Ib) exhibits the longest linker region, consisting of more than 40 amino acids. Nevertheless, the Ubx from proturan (SeUbx) and collembolans (FcUbx1, OcUbx) display markedly shorter linker regions, typically composed of only three to five amino acids. In certain species, this linker region may be absent. Remarkably, the sequences of the C-terminus display evolutionary patterns. In crustaceans, their Ubx (AfUbx, DmUbx) consistently maintain potential Ser/Thr (S/T) phosphorylation sites (Ronshaugen et al. 2002) (Fig. 6). In contrast, the insects, characterized by the loss of abdominal appendages in adult stages, feature a QAQA domain and poly-Ala stretches instead of phosphorylation sites (Galant & Carroll 2002; Ronshaugen et al. 2002) in their Ubx (DmUbx_Ib, DmUbx_IVa, DdUbx, MdUbx, AmUbx, and TcUbx) (Fig. 6). Interestingly, in the three groups of basal hexapods, proturans maintain short, cylindrical appendages on the first three abdominal segments (Headrick & Gordh 2009), diplurans possess a pair of cerci at the last abdominal segments (Headrick & Gordh 2009), and their Ubx (proturan SeUbx and dipluran CaUbx) have QAQA domains, poly-Ala stretches, and S/T sites. Meanwhile, in Collembola, which bears three types of abdominal appendages (Fig. 1C), the collembolans Ubx (FcUbx and OcUbx) lack poly-Ala stretches but contain the QAQA domains and a putative phosphorylated site Ser (S) in FcUbx (Fig. 6).

Genes with similar expression patterns usually participate in the same biological process (Ala et al. 2008; Bar-Joseph 2004; Bhar et al. 2013; van Dam et al. 2018). To infer the potential regulation of collembolan Ubx, we conducted Spearman correlation (*r* > 0.95, *p* < 0.01) analysis throughout embryogenesis (E_0.5d to E_9.5d) and identified a total of 113 genes with 287 transcripts coexpressed with *Ubx*, including the Hox gene *AbdA*, and phosphorylation kinase receptors (*ROR*) and phosphatases (*Ptp69D* and *Ptp99A*) (Supplementary Data 8). Given the sequence comparison and functional assays of FcUbx, we hypothesize that the collembolan Ubx may undergo direct or indirect regulation by protein phosphorylation or dephosphorylation during embryogenesis.

## Discussion

### Genes involved in the development of collembolan appendages

As a basal hexapod group, Collembola displays three pairs of thoracic limbs and three types of abdominal appendages. Investigating the genetic basis underlying these phenotypes could provide valuable insights into evolutionary loss of abdominal appendages in arthropods. In this study, we aim to demonstrate how to leverage developmental RNA-seq analysis and functional experiments to explore the evolutionary developmental mechanism of traits. To comprehensively identify genes involved in collembolan appendage development, we employed two distinct data mining approaches.

For the first approach, the “transcriptome-wide screening strategy”, we utilized the STEM method, a robust tool for identifying genes of predefined trajectory, including appendage development. We selected representative appendage formation stages, especially E_3.5d, when furca emerged (Fig. 1). This analysis identified several relevant appendage formation genes, such as *Notch, En, Scr, Antp, Ubx, Exd, Hth*, and *Lim1* (Table 1), which have been extensively studied in *D. melanogaster* for decades. Engrailed controls the segmentation of embryo (van de Heuvel et al. 1993), and Notch regulates the segmentation of leg (de Celis et al. 1998; Rauskolb & Irvine 1999); Hox genes Scr, Antp, and Ubx are required for the thoracic segment identity (Kaufman & Abbott 1984), Antp controls the formation of leg (Struhl 1982), and Ubx represses the abdomen leg (Castelli-Gair & Akam 1995; Gebelein et al. 2002). Exd, Hth, and Lim1 are essential for establishing the proximal–distal axis of limbs and regulating the formation of the coxa, femur, tibia, and tarsus (Costello et al. 2015; Ruiz-Losada et al. 2018; Tsuji et al. 2000). Among these candidate genes, we selected Ubx for downstream functional exploration for three reasons: (1) Ubx is one of the three Hox genes (Scr, Antp, and Ubx) identified in the STEM analysis (Table 1); (2) Ubx exhibits the highest expression level during the appendage maturation stage (Fig. 3E); (3) the gene expression profile indicates that Ubx may not repress *Dll* during the appendage formation stages (Fig. 3G).

The second approach, the “candidate-gene focusing strategy”, is designed by focusing on the pivotal *Ubx* gene and extracting coexpressed genes during embryogenesis. This analysis not only uncovered the canonical *AbdA* genes (Averof & Patel 1997; Casares et al. 1996; Konopova & Akam 2014) but also identified genes related to appendage formation (Supplementary Data 8), including *Lim1* and *DAAM*. In *D. melanogaster*, the deletion of DAAM results in abnormalities in actin filament structures (Barkó et al. 2010; Prokop et al. 2011).

These two synergistic approaches have elucidated a set of genes that have not undergone extensive investigation in arthropods except for *D. melanogaster*. Our analysis introduces new perspectives and broadens the scope of research directions. However, it is crucial to note that the transcriptomes we examined were derived from whole-mount animals, and the identified genes might also be involved in various biological processes, such as organogenesis and neurogenesis. Further research studies are necessary to verify the functional roles of these genes during collembolan appendage development.

### The evolution of functional domains in arthropod Ubx

Throughout its evolution, arthropod Ubx progressively acquired the capacity to inhibit *Dll* expression in the abdomen of insect adults, consequently resulting in the loss of abdominal appendages (Jockusch & Smith 2015). In this study, by integrating functional assays in collembolan Ubx, we reconstructed the evolutionary trajectory of functional domains in Ubx (Table 2): (1) The ancestral Ubx in Panarthropoda (onychophorans and arthropods) demonstrated a consistent DNA binding capability (Galant & Carroll 2002; Gebelein et al. 2004; Ronshaugen et al. 2002). However, their effectiveness in inhibiting the downstream target *Dll* varied among species (Table 2) (Galant & Carroll 2002; Gebelein et al. 2002; Grenier & Carroll 2000; Ronshaugen et al. 2002). (2) In Crustacea, the crustacean Ubx (AfUbx) is unable to inhibit *Dll*, and previous research has proposed that potential regulatory phosphorylation sites may be located in the C-terminus of AfUbx (Galant & Carroll 2002; Ronshaugen et al. 2002). (3) In Hexapoda, *Drosophila* Ubx (DmUbx_Ib) robustly represses *Dll* expression; the linker region and the C-terminus (including both the QAQA domain and the poly-Ala stretch) play crucial roles in this regulatory process (Galant & Carroll 2002; Gebelein et al. 2002; Ronshaugen et al. 2002). (4) Remarkably, collembolan Ubx shows the ability to inhibit *Drosophila Dll* expression, mirroring the capability observed in DmUbx_Ib (Fig. 5). Nonetheless, (5) unlike *Drosophila* Ubx, the linker region of collembolan Ubx is not essential for this repression (Fig. 6). Rather, (6) it appears that the functional repression domain and potential regulatory phosphorylation sites may be in the C-terminus of collembolan Ubx (Figs. 5 and 6).On the basis of these results, we summarize and propose a functional mechanism by which the collembolan Ubx suppresses *Dll* transcription that appears to be intermediate between that of crustaceans and insects (Table 2). Moreover, given the evidence of characters (QAQA domain, poly Ala stretch and S/T site) in the C-terminus of dipluran and proturan Ubx (Fig. 6), we speculate that the ancestral hexapod Ubx could bind and repress the expression of *Dll*. However, the scarcity of Ubx sequences in basal hexapods limits our understanding. Currently, only two complete collembolan Ubx sequences are publicly available (FcUbx and OcUbx), presenting discrepancies in sequence features (Fig. 6). With the advancement of genomic sequencing techniques, we anticipate that more Ubx sequences could be extracted from the genomes of basal hexapods. This, in turn, would facilitate the consolidation of sequence features in hexapods and enlighten the exploration of the evolutionary trajectory of functional domains in arthropod Ubx.

### The regulatory mechanism of collembolan Ubx on *Dll*

**Fig. 7:**
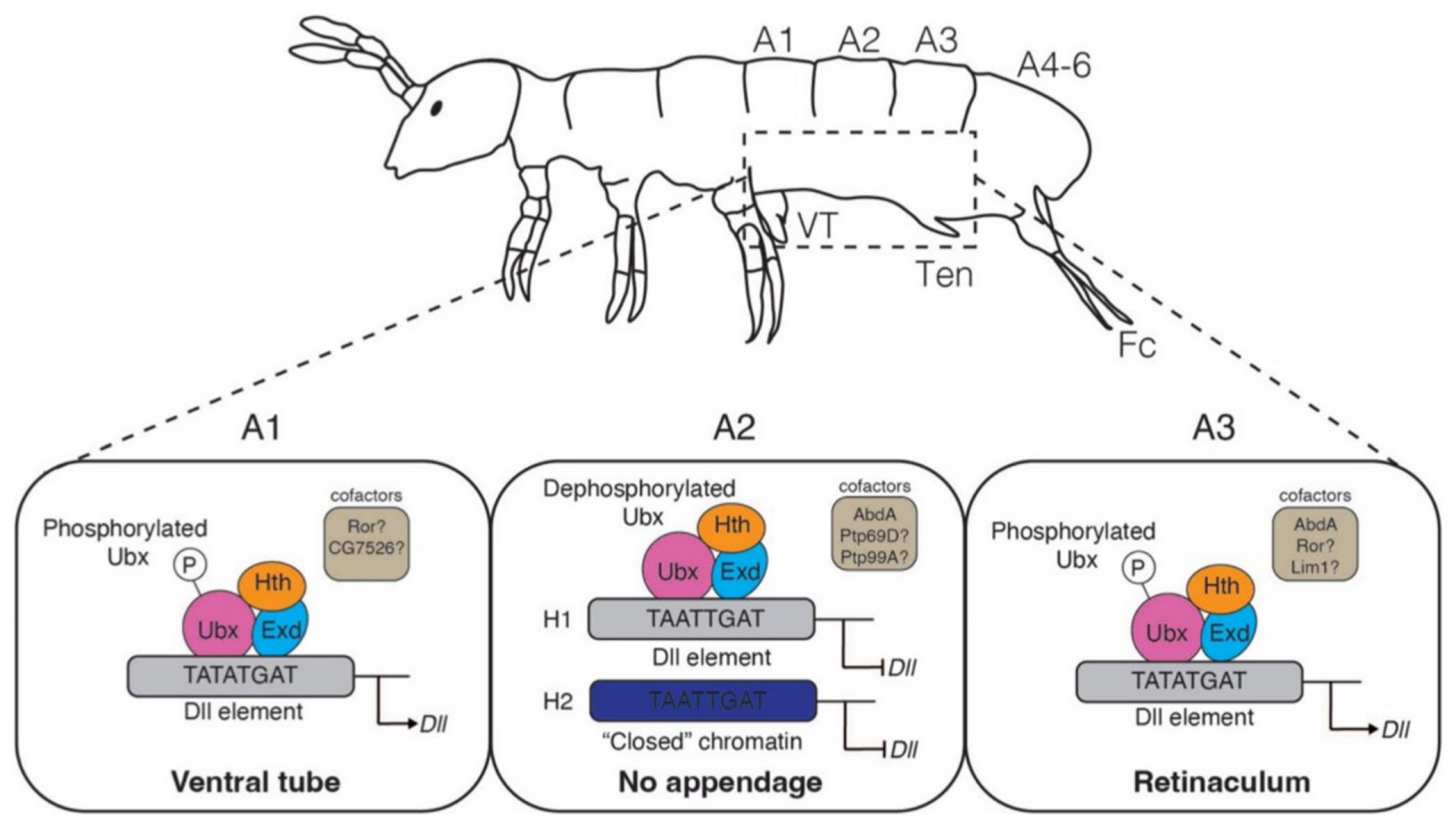
The proposed model for the regulation of collembolan Ubx on *Dll*. A schematic morphology of adult *F. candida*. The abdominal segments are shown as A1 to A6. VT, ventral tube; Ten, retinaculum; Fc, furca. The functional regulatory model of Ubx: in A1 and 3, Ubx might be phosphorylated, resulting in the derepression of *Dll* and facilitating the formation of appendages; Conversely, in A2, we hypothesise that (H1) Ubx could exert its repression function on *Dll* expression, or (H2) the chromatin of *Dll* regulatory region might be “closed”, thereby suppressing appendage formation

It is essential to highlight that the discussion on the functional evolution of Ubx primarily relies on the mechanism through which arthropod Ubx suppresses *Drosophila Dll*, and the mechanism of how arthropod Ubx (*trans-factor*) interacts with endogenous regulatory element of *Dll* (*cis-element*) remains underexplored. In combination with our bulk RNA-seq transcriptomic analyses and functional assays, we propose a model to discuss the mechanism by which collembolan Ubx regulates the abdominal segments: We hypothesize that Ubx uniformly binds and represses *Dll* expression during abdominal appendage formation across all segments (Figs. 3F, 4C, and 5). However, within each of the abdominal segments, collembolan Ubx is thought to implement its repression function in a spatially and temporally context-specific manner as depicted in our model (Fig. 7). During early abdominal appendage formation stage (E_3.5d) in abdominal segments 1 and 3 (A1 and A3), Ubx might be phosphorylated, potentially involving Ror (Table 1; Supplementary Data 2, 8), promoting the development of the ventral tube and retinaculum. The distinct morphologies of these abdominal appendages may be influenced by various cofactors, such as Lim1 and CG7526 (Table 1; Supplementary Data 8). For abdominal segment 2 (A2) and the appendage maturation stages (E_5.5d onwards), we hypothesis two scenarios: (H1) Ubx might be dephosphorylated by phosphatases Ptp69D or Ptp99A (Supplementary Data 8), enabling it to exert its repression function on *Dll*, resulting in the loss of appendages in A2, or (H2) it is also possible that the chromatin landscape of the *Dll* regulatory region may become inaccessible, preventing *Dll* transcription and appendage formation.

Despite the current scarcity of genetic tools in collembolans, the confirmation of our model demands incorporating cutting-edge experiments and functional genomics techniques. For instance, ChIP-seq (Park 2009) or CUT&Tag (Kaya-Okur et al. 2019) allows the identification of Ubx-*Dll* interaction in a genome-wide scale. Additionally, employing the CRISPR/CAS technology (Akinci et al. 2021) to manipulate the putative FcDll element would validate its role as a transcriptional regulatory element in collembolans. Furthermore, the incorporation of spatial transcriptomics (Marx 2021) and spatial ATAC-seq (Deng et al. 2022) will comprehensively map the intricate context of transcription factors and the regulatory landscape in each segment. These methodologies will empower us to investigate how Ubx works precisely, provide compelling evidence for holistic reconstruction of its functional evolution, and ultimately shed light on how arthropods lost their abdominal appendages during evolution.

## Supplementary Material

The online version contains supplementary material available at https://doi.org/10.1007/s00427-024-00718-0.

Supplementary Data

## Figures and Tables

**Table 1:** Genes classified in morphogenesis by BLAST2GO from cluster 42

Gene_ID	Gene_symbol	Orthologous in *D. melanogaster*	Molecular function
XLOC_013891	FBN2	CG7526	Embryonic limb morphogenesis
XLOC_001350	Exd	Exd	Somatic muscle development
XLOC_002645	Actin	Actin	Muscle contraction
XLOC_017923	BMI1	Psc/Su(z)2	Segment specification
XLOC_019142	GATA-3	pnr/grn	Anatomical structure morphogenesis
XLOC_016846	Wnt16	Wnt5/Wnt4	Wnt signaling pathway
XLOC_022735	En	Engrailed	Trunk segmentation
XLOC_011518	Ubx	Ubx	Specification of segmental identity
XLOC_011519	Antp	Antp	Specification of segmental identity
XLOC_015540	ROR1	Ror	Inner ear development
XLOC_005027	FOX	FoxF	Anatomical structure morphogenesis
XLOC_013406	MYO1E	Myo61F/Myo31DF/CG15831	Microtubule-based movement
XLOC_016945	Notch	Notch	Anterior/posterior pattern specification
XLOC_011524	Scr	Scr	Specification of segmental identity
XLOC_009469	Ski	CG7233/Snoo	Embryonic limb morphogenesis
XLOC_019633	Hth	Hth	Segmentation
XLOC_007689	LARGE2	No data	Muscle cell cellular homeostasis
XLOC_016637	Lhx1	Lim1	Anatomical structure morphogenesis

**Table 2:** Functional evolution of Ubx and schematic summary of the functional domains in Ubx of panarthropods

	DNA binding	Functional dissection of Ubx on *Dll* repression			
		Repression capacity of complete protein	Indispensability of linker	C-terminus	
				Repression domain	Phosphorylation site
				
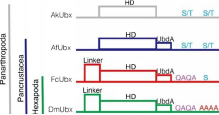	+	-	-	ND	+
				
	ND	-	-	-	+
				
	+	+	-	+	+
					
	+	+	+	+	-

+ strong phenotype,—no phenotype, *ND* no data

## Data Availability

The data supporting this study’s findings are available from the corresponding author upon reasonable request.

## Abbreviations

Ubx: Ultrabithorax
AbdA: Abdominal A
Exd: Extradenticle
Hth: Homothorax
Dll: Distal-less
Vt: Ventral tube
Ten: Retinaculum
Fc: Furca
An: Antenna
AnS: Antenna segment
Cllr: Clypeolabrum
DO: Dorsal organ
IcS: Intercalary segment
MdS: Mandibular segment
SEM: Scanning electron microscopy
STEM: Short Time-series Expression Miner
RPKM: Reads per kilobase per million
GO term: Gene Ontology term
KEGG pathway: Kyoto Encyclopedia of Genes and Genomes
IPTG: Isopro-pyl β-D-1-thiogalactopyranoside
GST: Glutathione S-transferase
DMXR: The transcription regulatory element of *D. melanogaster Dll304*
PFE: Putative FcDll element
EMSA: Electrophoretic mobility shift assay

## References

[R1] Akinci E, Hamilton MC, Khowpinitchai B, Sherwood RI (2021). Using CRISPR to understand and manipulate gene regulation. Development.

[R2] Ala U, Piro RM, Grassi E, Damasco C, Silengo L, Oti M, Provero P, Di Cunto F (2008). Prediction of human disease genes by human-mouse conserved coexpression analysis. PLoS Comput Biol.

[R3] Altschul SF, Gish W, Miller W, Myers EW, Lipman DJ (1990). Basic local alignment search tool. J Mol Biol.

[R4] Angelini DR, Kaufman TC (2005). Comparative developmental genetics and the evolution of arthropod body plans. Annu Rev Genet.

[R5] Angelini DR, Kaufman TC (2005). Insect appendages and comparative ontogenetics. Dev Biol.

[R6] Averof M, Patel NH (1997). Crustacean appendage evolution associated with changes in Hox gene expression. Nature.

[R7] Bairoch A, Apweiler R, Wu CH, Barker WC, Boeckmann B, Ferro S, Gasteiger E, Huang H, Lopez R, Magrane M, Martin MJ (2005). The Universal Protein Resource (UniProt). Nucleic Acids Res.

[R8] Bar-Joseph Z (2004). Analyzing time series gene expression data. Bioinformatics.

[R9] Bar-Joseph Z, Gitter A, Simon I (2012). Studying and modelling dynamic biological processes using time-series gene expression data. Nat Rev Genet.

[R10] Barkó S, Bugyi B, Carlier MF, Gombos R, Matusek T, Mihály J, Nyitrai M (2010). Characterization of the biochemical properties and biological function of the formin homology domains of Drosophila DAAM. J Biol Chem.

[R11] Berger MF, Badis G, Gehrke AR, Talukder S, Philippakis AA, Peña-Castillo L, Alleyne TM, Mnaimneh S, Botvinnik OB, Chan ET, Khalid F (2008). Variation in homeodomain DNA binding revealed by high-resolution analysis of sequence preferences. Cell.

[R12] Bhar A, Haubrock M, Mukhopadhyay A, Maulik U, Bandyopadhyay S, Wingender E (2013). Coexpression and coregulation analysis of time-series gene expression data in estrogen-induced breast cancer cell. Algorithms Mol Biol.

[R13] Brower DL (1986). Engrailed gene expression in Drosophila imaginal discs. Embo j.

[R14] Brown JB, Boley N, Eisman R, May GE, Stoiber MH, Duff MO, Booth BW, Wen J, Park S, Suzuki AM, Wan KH (2014). Diversity and dynamics of the Drosophila transcriptome. Nature.

[R15] Browne WE, Patel NH (2000). Molecular genetics of crustacean feeding appendage development and diversification. Semin Cell Dev Biol.

[R16] Budd GE, Telford MJ (2009). The origin and evolution of arthropods. Nature.

[R17] Buffry AD, Kittelmann S, McGregor AP (2023). Characterisation of the role and regulation of Ultrabithorax in sculpting fine-scale leg morphology [Original Research]. Front Cell Dev Biol.

[R18] Casares F, Calleja M, Sánchez-Herrero E (1996). Functional similarity in appendage specification by the Ultrabithorax and abdominal-A Drosophila HOX genes. Embo j.

[R19] Castelli-Gair J, Akam M (1995). How the Hox gene Ultrabithorax specifies two different segments: the significance of spatial and temporal regulation within metameres. Development.

[R20] Cohen SM (1990). Specification of limb development in the Drosophila embryo by positional cues from segmentation genes. Nature.

[R21] Cohen B, Wimmer EA, Cohen SM (1991). Early development of leg and wing primordia in the Drosophila embryo. Mech Dev.

[R22] Cohen B, Simcox AA, Cohen SM (1993). Allocation of the thoracic imaginal primordia in the Drosophila embryo. Development.

[R23] Conesa A, Götz S, García-Gómez JM, Terol J, Talón M, Robles M (2005). Blast2GO: a universal tool for annotation, visualization and analysis in functional genomics research. Bioinformatics.

[R24] Costello I, Nowotschin S, Sun X, Mould AW, Hadjantonakis AK, Bikoff EK, Robertson EJ (2015). Lhx1 functions together with Otx2, Foxa2, and Ldb1 to govern anterior mesendoderm, node, and midline development. Genes Dev.

[R25] de Celis JF, Tyler DM, de Celis J, Bray SJ (1998). Notch signalling mediates segmentation of the Drosophila leg. Development.

[R26] Deng Y, Bartosovic M, Ma S, Zhang D, Kukanja P, Xiao Y, Su G, Liu Y, Qin X, Rosoklija GB, Dwork AJ (2022). Spatial profiling of chromatin accessibility in mouse and human tissues. Nature.

[R27] Durston AJ, Jansen HJ, In der Rieden P, Hooiveld MH (2011). Hox collinearity - a new perspective. Int J Dev Biol.

[R28] Ekker SC, Young KE, von Kessler DP, Beachy PA (1991). Optimal DNA sequence recognition by the Ultrabithorax homeodomain of Drosophila. Embo j.

[R29] Ernst J, Bar-Joseph Z (2006). STEM: a tool for the analysis of short time series gene expression data. BMC Bioinformatics.

[R30] Ernst J, Nau GJ, Bar-Joseph Z (2005). Clustering short time series gene expression data. Bioinformatics.

[R31] Faddeeva-Vakhrusheva A, Kraaijeveld K, Derks MFL, Anvar SY, Agamennone V, Suring W, Kampfraath AA, Ellers J, Le Ngoc G, van Gestel CAM, Mariën J (2017). Coping with living in the soil: the genome of the partheno-genetic springtail Folsomia candida. BMC Genomics.

[R32] Fountain MT, Hopkin SP (2005). Folsomia candida (Collembola): a “standard” soil arthropod. Annu Rev Entomol.

[R33] Galant R, Carroll SB (2002). Evolution of a transcriptional repression domain in an insect Hox protein. Nature.

[R34] Gao Y, Bu Y, Luan Y-X, Yin W-Y (2006). Preliminary observation on the embryonic development of Folsomia candida (Collembola: Isotomidae. Zool Res.

[R35] Gao Y, Bu Y, Luan YX (2008). Phylogenetic relationships of basal hexapods reconstructed from nearly complete 18S and 28S rRNA gene sequences. Zoolog Sci.

[R36] Gaunt SJ (2015). The significance of Hox gene collinearity. Int J Dev Biol.

[R37] Gebelein B, Culi J, Ryoo HD, Zhang W, Mann RS (2002). Specificity of Distalless repression and limb primordia development by abdominal Hox proteins. Dev Cell.

[R38] Gebelein B, McKay DJ, Mann RS (2004). Direct integration of Hox and segmentation gene inputs during Drosophila development. Nature.

[R39] Geyer A, Koltsaki I, Hessinger C, Renner S, Rogulja-Ortmann A (2015). Impact of Ultrabithorax alternative splicing on Drosophila embryonic nervous system development. Mech Dev.

[R40] Giribet G, Edgecombe GD, Wheeler WC (2001). Arthropod phylogeny based on eight molecular loci and morphology. Nature.

[R41] Grenier JK, Carroll SB (2000). Functional evolution of the Ultra-bithorax protein. Proc Natl Acad Sci U S A.

[R42] He JM, Zhu H, Zheng GS, Liu PP, Wang J, Zhao GP, Zhu GQ, Jiang WH, Lu YH (2016). Direct involvement of the master nitrogen metabolism regulator GlnR in antibiotic biosynthesis in Streptomyces. J Biol Chem.

[R43] Headrick DH, Gordh G, Resh VH, Cardé RT (2009). Encyclopedia of insects.

[R44] Hughes CL, Kaufman TC (2002). Exploring the myriapod body plan: expression patterns of the ten Hox genes in a centipede. Development.

[R45] Hughes CL, Kaufman TC (2002). Hox genes and the evolution of the arthropod body plan. Evol Dev.

[R46] Jockusch EL, Williams TA, Nagy LM (2004). The evolution of patterning of serially homologous appendages in insects. Dev Genes Evol.

[R47] Jockusch EL, Smith FW, Wanninger A (2015). Evolutionary developmental biology of invertebrates 5: Ecdysozoa III: Hexapoda.

[R48] Kanehisa M, Goto S (2000). KEGG: kyoto encyclopedia of genes and genomes. Nucleic Acids Res.

[R49] Kaufman TC, Abbott MK, Malacinski GM, Klein WH (1984). Molecular aspects of early development.

[R50] Kaya-Okur HS, Wu SJ, Codomo CA, Pledger ES, Bryson TD, Henikoff JG, Ahmad K, Henikoff S (2019). CUT&Tag for efficient epigenomic profiling of small samples and single cells. Nat Commun.

[R51] Kim D, Pertea G, Trapnell C, Pimentel H, Kelley R, Salzberg SL (2013). TopHat2: accurate alignment of transcriptomes in the presence of insertions, deletions and gene fusions. Genome Biol.

[R52] Kolde R (2012). Pheatmap: pretty heatmaps R Package Version.

[R53] Konopova B, Akam M (2014). The Hox genes Ultrabithorax and abdominal-A specify three different types of abdominal appendage in the springtail Orchesella cincta (Collembola). EvoDevo.

[R54] Krogh PH (2009). Toxicity testing with the collembolans Folsomia fimetaria and Folsomia candida and the results of a ringtest. Miljøstyrelsen Environmental Project Miljøprojekt No 1256.

[R55] Kurata S, Go MJ, Artavanis-Tsakonas S, Gehring WJ (2000). Notch signaling and the determination of appendage identity. Proc Natl Acad Sci U S A.

[R56] Langmead B, Salzberg SL (2012). Fast gapped-read alignment with Bowtie 2. Nat Methods.

[R57] Liang Y, Xie W, Luan YX (2019). Developmental expression and evolution of hexamerin and haemocyanin from Folsomia candida (Collembola). Insect Mol Biol.

[R58] Luan YX, Mallatt JM, Xie RD, Yang YM, Yin WY (2005). The phylogenetic positions of three Basal-hexapod groups (protura, diplura, and collembola) based on ribosomal RNA gene sequences. Mol Biol Evol.

[R59] Luan YX, Cui Y, Chen WJ, Jin JF, Liu AM, Huang CW, Potapov M, Bu Y, Zhan S, Zhang F, Li S (2023). High-quality genomes reveal significant genetic divergence and cryptic speciation in the model organism Folsomia candida (Collembola). Mol Ecol Resour.

[R60] Marx V (2021). Method of the year: spatially resolved transcriptomics. Nat Methods.

[R61] Matsuda R (2017). Morphology and evolution of the insect abdomen: with special reference to developmental patterns and their bearings upon systematics.

[R62] McGinnis S, Madden TL (2004). BLAST: at the core of a powerful and diverse set of sequence analysis tools. Nucleic Acids Res.

[R63] McIntosh BB, Ostap EM (2016). Myosin-I molecular motors at a glance. J Cell Sci.

[R64] Mead TJ, Martin DR, Wang LW, Cain SA, Gulec C, Cahill E, Mauch J, Reinhardt D, Lo C, Baldock C, Apte SS (2022). Proteolysis of fibrillin-2 microfibrils is essential for normal skeletal development. eLife.

[R65] Monteiro AS, Ferrier DEK (2006). Hox genes are not always colinear [review]. Int J Biol Sci.

[R66] Natori K, Tajiri R, Furukawa S, Kojima T (2012). Progressive tarsal patterning in the Drosophila by temporally dynamic regulation of transcription factor genes. Dev Biol.

[R67] Noyes MB, Christensen RG, Wakabayashi A, Stormo GD, Brodsky MH, Wolfe SA (2008). Analysis of homeodomain specificities allows the family-wide prediction of preferred recognition sites. Cell.

[R68] O’Day KE (2006). Notch signaling and segmentation in Parhyale hawaiensis.

[R69] Palopoli MF, Patel NH (1998). Evolution of the interaction between Hox genes and a downstream target. Curr Biol.

[R70] Panganiban G, Irvine SM, Lowe C, Roehl H, Corley LS, Sherbon B, Grenier JK, Fallon JF, Kimble J, Walker M, Wray GA (1997). The origin and evolution of animal appendages. Proc Natl Acad Sci U S A.

[R71] Park PJ (2009). ChIP–seq: advantages and challenges of a maturing technology. Nat Rev Genet.

[R72] Passner JM, Ryoo HD, Shen L, Mann RS, Aggarwal AK (1999). Structure of a DNA-bound Ultrabithorax-extradenticle homeodomain complex. Nature.

[R73] Peel AD, Chipman AD, Akam M (2005). Arthropod segmentation: beyond the Drosophila paradigm. Nat Rev Genet.

[R74] Prokop A, Sánchez-Soriano N, Gonçalves-Pimentel C, Molnár I, Kalmár T, Mihály J (2011). DAAM family members leading a novel path into formin research. Commun Integr Biol.

[R75] Rauskolb C, Irvine KD (1999). Notch-mediated segmentation and growth control of the Drosophila leg. Dev Biol.

[R76] Reed HC, Hoare T, Thomsen S, Weaver TA, White RA, Akam M, Alonso CR (2010). Alternative splicing modulates Ubx protein function in Drosophila melanogaster. Genetics.

[R77] Ronshaugen M, McGinnis N, McGinnis W (2002). Hox protein mutation and macroevolution of the insect body plan. Nature.

[R78] Rozewicki J, Li S, Amada KM, Standley DM, Katoh K (2019). MAFFT-DASH: integrated protein sequence and structural alignment. Nucleic Acids Res.

[R79] RStudio (2020). RStudio: integrated development for R.

[R80] Ruiz-Losada M, Blom-Dahl D, Córdoba S, Estella C (2018). Specification and patterning of Drosophila appendages. J Dev Biol.

[R81] Ryoo HD, Marty T, Casares F, Affolter M, Mann RS (1999). Regulation of Hox target genes by a DNA bound homothorax/Hox/extradenticle complex. Development.

[R82] Slattery M, Riley T, Liu P, Abe N, Gomez-Alcala P, Dror I, Zhou T, Rohs R, Honig B, Bussemaker HJ, Mann RS (2011). Cofactor binding evokes latent differences in DNA binding specificity between Hox proteins. Cell.

[R83] Struhl G (1982). Genes controlling segmental specification in the Drosophila thorax. Proc Natl Acad Sci U S A.

[R84] Timmermans M, Roelofs D, Mariën J, van Straalen NM (2008). Revealing pancrustacean relationships: phylogenetic analysis of ribosomal protein genes places Collembola (springtails) in a monophyletic Hexapoda and reinforces the discrepancy between mitochondrial and nuclear DNA markers. BMC Evol Biol.

[R85] Trapnell C, Roberts A, Goff L, Pertea G, Kim D, Kelley DR, Pimentel H, Salzberg SL, Rinn JL, Pachter L (2012). Differential gene and transcript expression analysis of RNA-seq experiments with TopHat and Cufflinks. Nat Protoc.

[R86] Tsuji T, Sato A, Hiratani I, Taira M, Saigo K, Kojima T (2000). Requirements of Lim1, a Drosophila LIM-homeobox gene, for normal leg and antennal development. Development.

[R87] Tully T, Potapov M (2015). Intraspecific phenotypic variation and morphological divergence of strains of Folsomia candida (Willem) (Collembola: Isotomidae), the “standard” test springtaill. PLoS ONE.

[R88] UniProt Consortium (2018). UniProt: the universal protein knowledgebase. Nucleic Acids Res.

[R89] van Dam S, Võsa U, van der Graaf A, Franke L, de Magalhães JP (2018). Gene co-expression analysis for functional classification and gene-disease predictions. Brief Bioinform.

[R90] van de Heuvel M, Klingensmith J, Perrimon N, Nusse R (1993). Cell patterning in the Drosophila segment: engrailed and wingless antigen distributions in segment polarity mutant embryos. Development.

[R91] Waterhouse AM, Procter JB, Martin DMA, Clamp M, Barton GJ (2009). Jalview Version 2—a multiple sequence alignment editor and analysis workbench. Bioinformatics.

[R92] Weatherbee SD, Carroll SB (1999). Selector genes and limb identity in arthropods and vertebrates. Cell.

[R93] Wickham H (2016). ggplot2: elegant graphics for data analysis.

[R94] Wu J, Cohen SM (1999). Proximodistal axis formation in the Drosophila leg: subdivision into proximal and distal domains by Homothorax and Distal-less. Development.

[R95] Yuzuki D (2015). BGISEQ-500 debuts at the International Congress of Genomics 10.

