# Supplementary material for: The functional evolution of collembolan Ubx on the regulation of abdominal appendage formation
Source: Dev Genes Evol. Author manuscript; Available in PMC 2024 Sep 20. (PMC7616481; doi:10.1007/s00427-024-00718-0)
Supplement: Supplementary Data [file EMS197062-supplement-Supplementary_Data.zip › 6-Genomics sequences of F. candida Dll.docx]

>dll_combined_gDNA_(F._candida) ATAAAAATAATACAATCGTGACACATTTCATAACTGAAATGTGCGTACAAATAGTTAAACAAAATGGATAGCTGTAGTATTTAAGTACAAGTTTGCTGACAATACCATTTTGAAATTTTAACAGACAATTTTCTACAAATCTCCAGTTTTTAGGTGACAACTGCACTTGTTCACAACAAAGGACACGAATGACAATGGATTCATCGCATCCAACACCGCAAGGACCACCGTGAAATCGGAAATCACTTCACGCCAACTTTGCTCAAGCCTTTCTTTCTCGAGATGACAAAAAAGTGAGCGAGATTCTCAACAAACAATGAGAAATGTGTGTGTTATATATTTGACGAACTTTAATCTTCATTGTGAGACTTTGGCCCAGCTTGGGTCCGTTGTTTTCACATAAAATCGGTTATCGTAACTTTGTATGTTTATGCGAAAGGTGAAATATTGGCTGAAATATAATTCACGAGTTACATATTTATGTAAGTTTTAAGGATTCGTTTGGTCTGGTGCATTCAACTTTAGTGCAGTTGATTATTACTTCATAATTCATCCACTAATTACACGAGGAGGATGTTTGTTCTCATATTAAAACATTATTAAGTTTCAATTACACATACCGTCTTACATTTCGCCAATTACTGTAATAACACACGTGTCCATAAGTACTCAACTTCATACTTCATATATGCATGCATGTATGATGTATGTAAGTACCTCTTGAATTCAATAAAGTTGGAAATATTACAAACATGTACATATCTACATGCACATGTAGCAACTGGCAGAAAATAAGTCGGGTTGAAGTAATCCAAGCATATCTTTCATTAAGTAAAGTTATTGAGTACCCTGGCTTCCGAAACGTGTATGTAGGTGGATAATTGACCATGACGTACGAATACTTTCTCTGCGGTGATATATAGTAGTCATTTTTGAAAATATAATGTACGCACTGTGCCACTCGTCATATTGGGTCCAAGGGAGGAGCAATTATTTTGCGAGTGGCATCCCATCCAACGAAATGCCACCCTTCCAACCGGAAAAGAAAGAAATATGAAATGTGTAGGCTTGCTCAATCAATTCGCGACATGTGGAATTATTAGCATATTTATTAATTAGTGACCACACCCACGCCAAGCTACCCAGGCACAGGAATGACAACATGTACCAGAACTGATCCAGTATGGAAAAGGCAAACAGAGAGAGGGTGACAAGACAAACCCCTCCGGAATACAATACGTGATGTGTACAGACATACACGCAATATGGACGACTTGGTATGTATGTCGACGTGATGAAACAGAAAACTGAAATGTGACAAGAGGATTCTCGATATAGGCACTTGGTGTTGTTTGAAATGTGAGCAATATGGCAAATTAATTAGTTTCAGTCGCCACCATCCACTCAGCCAATCCACTCAGAATGGAGGTCTCCCAACATTTTTCACAGATTATGGCCAAATAATGAATTGATCAGAAATTTAATTAATAACAAATTTGCAAAAGATATTCTCAGCCAATGTGCAGGTTTGGCAGGGATATTTGCATTTGAAGAGGAGAAAACGCACAGTTAATTTTGAAAAGTGTAAAATTTGTCACCATTAGGGCGCGCTTTCTCCTCTTTTGATGCAAATATCTCTGCCAAACCTACGCGTTGAGAGATATTGTCTTTTACAACTTTCATATCACTTAACTTTGTGATCTATTGAGACTAATTTGACCAAAATCGGAGAGCATACGCTGGAAAACCTCCATTTTGAATGCGTAAAACTTTCACCACTAAGCGATTGATTCATCATTGTATGTTATGTAGCTTTTTTCCCTCCACAATAGCAATAATACTAATTCTGAGAGGCTCTTATGCCCATTTCGACTGGGGAGAGGTCGGCTATGATCATAAAAATATGTCATTCGATAATAATGGTAAAAAGACCAATTTATCCATCCACTCACAATAAATCCCGGCTCTTATAACACCAACCAACACCACACGGCCATGAGGAAAGTACAACAACAACAGGACGGGCTGGCATTCGAGGTGTGCCTTTACCCAACACCACAGGCAATTTATCAACTTAACAGGAGACAAGAATAAAAGAGGAAAGCAAGCAGGCAGAAAATTGGGCAATTTAGCGAATTCACCCCAATTCGGGTAATTGAATTGTCTAACAAACACTGACACTACGTAGCAGCCAAAACCCAACCGTTTCAACCTCTCTTGAATTATTTTCTCTACAATCTCGCAGAATTTCCTTCCCCAGAGCAATTTACAGAAATCTAAGAAAAGCAACAGTGGTGCAGTGTCGCGAATTATTTTATTAATAAAAGGACGCCATCGGTGCGTTCAGCAATTCTGCCACAGTTTTCGTGTCAGATCATATTTTATCTCGAGTACTTATTTACCACGACATTACTCACGATTTAATTGATCAAACTTCATTGTCCAACTTTTTCATGGCGTTTATGCATCAACTCCCAACTTTCCCCGCAACTTTCTCCTATTTCGTTGTTGGGAGAAGCTGTTTTTCGAGAAAATTGAGGTCCTAGTGGAATCGTTCCTCTAATATTTTATTCATCCTTCATCTATGTTCTTGGAGAAAGCCACAGGTAAAAGTGGCGGAATAACAGGAGGAGAAGCATGTTTCACCCACCACGGGCCAAACAAGTGCTAAATAAGTTATAAACTAAATTGCACATTTATCCAGCCCAAACTTTCCCGTAAACGGAGCTTATTCATCCATCCCCTCTCATTATTAGGGAAATAATAATTTTATTTTTCTCAATTTAAGAATTTCCCTTCCCTTCATTCGTGTCGACGAGGTGAACATTTATTTCTTTAATTCTTAAATCCCTTCAATTTCCTAATCCATAACATTACTTAAAACGTTTAATTGTGTTTAAATACAAGTCTTTAAATCTCTCAAGGACTGGTCAAATTTCGAGTTTTAGTTTCATTTTAAAATTGCTATGTATGTACCACCAGAAAATCGGCAGGATTATTTGATACACATTTCTAAAAACAAGAGATTTTCTATTTCGACACGATTTTCTGGTTGTGCTCGATTTGATTACAGAATCGAACGATGTAGGCAAGTCTTTGAATAGCGAAATCTTTCGACTCGGAGTCAAAAGTGGTTGGTGGCGTTGGAGGAGGTGGCCTGCGAGGCGTAATCCAGCCCAAGCGTTTCTGAGGCGGAGTCGGAAAACTACCCATCAGCCCAGCACGACCATAGCGTCAGAGCCATAGGAAAAAGGTACCTTTGCTGGGGGTAGTTTAGCATAGCTTTAGTTTAGTATGGGGAAGGTAAGGTTGGATTTGGTTTGGTAAGGTGAGGAATGATTCCCATCCGCAATTACAGTTTCACGAGGGCAAGCGAGCTGACGAGATCACGACTCACCTCACTTATTTACTCCCAACCAACTTCTTGTGAAAAAAGTCTCAATAAATTTATACACGGATCAGCCTCGCTTTTAGATACGATCTGTCATTTGTGGTTTCACAGATAAGCTGCACAAAGATTCCCAGCCCAACGCTTAATGGGCAATTTGTCCACGCTGCTCACTGCTCCTCTTCTTCTAATCCAGACCAATTTGTGGTTTATACGCGTCTCAAATGGTATCGGGAAAAGTGACACAAAACTAGAAACCTAGTTATCTAAAAGATAAGGACACGGAGGAAAAAGATAAGGCTGGAATTTTTTTGGGGGAAATGCAGCATTGAAAAGTGACTGAATTTTACGGAAAGTAAAAATAAAGCCGGCATTCAACAGTTTAGAGTTTTTGTGGTGCACTCATTAAGTGTAATTAGTGAAATTACTCTCACGTGCGCGTACCAACGAACGAATGAGCCCTTCTTCTTCGCCTTACTTCCGCTTGGGTTGAGTGCAGGAAGTGAAAGTTATTTATGAAATAAAACCTCGATTTTGACAAGAATATTGTAAAAGTAGAATTTTTCGAAATTTCAAAGACAACTTGACACGACTTAAGTAAGCTTGATATGGAACTACAGAGTGAGCTGAAACTTTGCAATTTGAATATCCTAGTGACTATTGCGGATAGTGAACATTTGATCAGCTAGAAATAAAGAAGGATTCCACGGGTGTCAGTTTACCCCCGATCGAACAAAACGTTAATCTCTGGGACTGAAACAGCCTGTCCCCCAGAGAAATTTCCAGTACCACGACCCGTCACACGACGTCGACAACAAGTCAGATTTATTTTGTCGTGATAACCTCGTTACAAACATTCTGAATTTCGGGGAATTAAACCCGTTTCCGGGCCTGGGACTTTCGTCACCGGGACCCGACGGGTTAGACGAAATGCAACACCAGGGGTCCTCGTATAGAGCGGCCGCGGCTGCTGCGGCCGTTGCTGCTTATCAAGAGCAGAACTACAGGTCAGGTGGATATCCCTTCCCCCCACAAAACCCGTACGGGTATCATCTCGGAAACTACCCGCCTCAATGTTCCTCCCCTCCGAAAGATGGTGAGTGACCGAATTTTTTAATAAGTTATTATTGTGACACGAGAATTGTAAAAATGTATTCACAGATAGTCAGGCATCGGAGAGAAATTCATTAGGGCGTACAAATTTTGGTGGTATAATGGACATTAATTGTCAGAAATTCTAAATAGTCAGATTTATTTATCGAACATTGCACTCCACAAGATCCTTTCTTATCATATTTTGTATTGCTGGTCATGATGCAATATCAATATAATCATATCACAATATATGTATAAGTCTGCATGGATTTTCTGCAGAACATTAATCCATTCTCACTCTTGTCAGTTGTAATGTTGTAAATGTATGTACACGCAAGTGTCTAAGAAAATGTGTCTCATAAAATCTCGACATTCCCCAAACAGGCATCGATGCTACCATGATTATTATTTTATCCTGGGCTTCCTTTTAAGTACTCGAATATTTTATGCATACGCTTTTTCCGGACATAATTTCTCCCATCGTGAATAATTAAGCATGTTTAACCAACTATTTAAACAAAAGCCTGGAATCATGCTCCCTACATTTATAAATATCTACTTGAGCCTAGTTAACTCTTTCACAAGGGCTGCAGGAGGGGTATCACGTATCCGACCGAATTTCCATCAGATAAACGACCACAAGAGCAAAATGGGTGCTTGCTAACTCGCTCATCCTATTAACCCGTATTACGTATTCATGATACCCGGTTGTTTGTATTCCTGGGAAAGGTTTACGAGCTCTTGTGTCACATATGTAGGAACCACCAAGGAGCTGTTGACATTAGCGGTTGGTGGGATTTCCTTCCTACGAGTGCTTAGTTAGCATGGCATGACAGTCTGTTTACTTTGGAGCTTATTACTGGGAAATATTTGAGCAAAATTGCAACTGCGAAGCCAAGAATGAAATGAAGTAACAAACCGTGAAAAGAGCAATTTTAGAGATAAAATAAAATAAAATAAATAAACGGGTTCGTATTTTTCGGCTTATGTACATATTCTTAGCTACATGTTTATGTAGTCGGCGTATTTTTGGTATTCAGCAGAACCTGAGTAAACAGAAAATAATATATAAAATAACAATGATTACGCAAAATCATTAAGTAGAAAATTTCCACGGCTCGCAGTCATAGTTGGTAAAATGAAATTGGTCTGGTTTCGTGTACAGCATGGAATAATTTAGTATGACGTGTTGAATAAGGAATTGAGGGCTCAAGTATTTCACTCTGCATTCACATTCATCATATCAAATGACACATGAAACTCTGGACAAACTGCGTACATGATCCCATCGTTAGTTGGTGAAGAACCAACCTGATCCACATCCCCCTTCCCAGCGGGACCAATAATAATAACTCGTCGCGTATGTATACCTGGTGGCCAGAATTGAATGACAATGAAACAAGGTCCTTGAGGAGTTGGGTCATGTTCGGGTCATCTGGTTTAAATGGGATCAAGTGTCAGACATACGAACTCACCCCCACCTCTCCGCCTGTACTGATCTCTGCCCTAATAAAATCGGGCATATCGTGTGCATACGTCCATTTGTGTTAAGACGGGCATAGATACGTGAAAATGGGAATAATTGTGATTTTCAGAGCCGAAAGAGGAGCCGGGTGGAATGCGGTTGAACGGAAAAGGGAAAAAGATGAGAAAACCACGGACAATTTATTCCTCCCTACAATTACAGCAGCTCAACCGCCGCTTCCAGCGCACGCAGGTTCGTTGCTTAATTATTTGGTTAATTAAACACTAATTAAAAATATGTAATAATGACTTAATTTCATTTTTGCAGTATTTAGCTCTACCTGAACGCGCGGAACTCGGTAAGAACCATCTTTATTTGCAATTAGTATTTGTAGTACAGTTTAAAATCTGGTAGAGTCACTAGTTTGGCATTAAATTCCTACCACATTGGTAGTAGTTTTGTTTCCCAGTGGTAAATTTTTCCTTATCACAACATTGCTAAACGGTAATGCCCGTGTTATTAGAGCCACATACGTACTCCGGTCAGTAATGATTTAATACCAGTTTTTTAAGAGTATATGGATAAAATATTAGCAAAAAATTCTGATCAAATTTCGATTAAGATTCCCTGTGCAATATTAATAACGCATCATTCTTATTTTATTTCAGCTGCCTCTCTTGGTCTCACACAGACACAGGTTTGCAACCCTTCCGTATTTTTGGAATTTTTACCTCATAAATACTACGAGCAATTCGGCTTGTTGTATCGCATATATACTTAAATGGTTGAGTAGATTTTACAGCAAATCATATCGTGGAGGAAAATATTATTGTATGAAATCTGAAAAGCACATCATCTCGAATATCAAAGAATTCCCTTTCGTCACCTTTTAAAAGAAGTATTAAGACTCCCAATAAATAAATCTCAAGTGCTCTGAATGTGTAAGGATCGTCATACGTGGTGCTGTTCCTCCTTCACTTCTTTTCCTCATCTTTTTCCCTCATGTCCCAATCTGCTCACCTTTCCTCACGTGAAGTTTGGCCGGAACGGAAATATCAAAGAAGCAAGACAGCCAAGATATGGGACTGAAAGAAGTTGAGAATTTAGAATCTCTTAAAGCAGCAGGGGACCATTCTTCAATTAGACACGGTTCTCCCTATTTCACTCGCCCTCTGTGCTCCACATTCCCTCATCTTCTCCTTCAGACTTATCACTTTTCGGTAGCTATTTCCTTCGTCCTAGGTGAGGTTGCAGACCTCACGAAATCTGAGACATTTCCACTTTTTACTACAAGTATGTACTCGAAAAAACAAATTTTTACACAGATTTTGAAGCCGAGAGCAATAAATTTCCTGGATAAAGATAACACAAAAACAATGCTGCTGCTCCTCTTGGTGGTAATAAAGCGAGAGATTAATTGCTTTCGGCTTGATATTTCACATTCTGTTGAGCACAGCAGTAGTTCACACAAACACAATGCGATATACGGACATAAATTCTGTTTCTAGTGAGTTATTCTCTACGTTCAAGTGTCCTTCCTGTCCCAGGTCGCACATACATAAGCATAAGTATTTAATAAATAGTAAAAAAGACAGCAGTACGGGTACCCTTTTTACTTTCCTTTTCTTAATAAAGTTCTTTTTTGCCGTAGTGTTGTACTCACCAAGTCAGAATTGAGAGCATGTCCATCCACATATCTGTAGGCAGGGAGAGGAGGAAATACAGGAACAATAGGCTGTGAGCAGGACAAGATAATTTATTTTGAGGAAGCCCGAACGGTAGCTAATCCAAGATAATTTATAAATTAGTCGTGAAGAGGGAGAAATGGAAAAAGCCTCATCATTATTTAACTTGTACAAGCAGAGAAAATCCATTGTGCTCCATTTAAAGCGTAGCGTATTAAAAGAGTCGAAGAGATGCAGCACAAGTAGTTCACTTCTTCTCCTTCTTTGGGGAAGAATGGATTAGATTTGCACGGTTGAGGTTGAGGTTGACGGTAAACAAGTGTGACATGATGTGATTGTGAGGACCTAAGTGATACCCCTCGAGCACAAGCAACCACATTTTTAACCCTTTTTTACTGTACCCCAGGAATTCCTTAAAGGATTGACAAAAAATAATGCATCGCAATTTATTTTACGATAATATTTGCGTAGGACTTGTGCACATTATGAAAATGTGGATGATGGGTGCATATTAATCGCACATGCACCCACTCCTTTCCAATGTTGTTGTTGTTTAGAATGCAGTTTGAAACAACACATTGTTGTCTGTGAGTGAGTGGAGATGGTGAATGTTTTAGCAGTTGGTAATGGAAGGGTTCTTCTACTCATTTGGTGTGGTTATTGGTGCCCCGGCCGAGTTGATGATGGGGCCGGTCTGACACCTTAGAATTAAAAGCAGCAGGGGAGCAGCTTGTCACTGTCAAAGTCTTCATGTCTTGCACTACACCAAACCAAAAGGTAATGATAAAATGCAATGTTGCGACTTTCTTCCTTCAAATGTGTGTCATGAATGAAATCCTTGGAACAAGCCATGAGACAGGGAAGTGTACATACATTCGATCGAGGGGTTCAAGTTTCTTAAACTTTTATAGAAATTGCAGAGTGGAAAGAAATGGTGAATCCCGTTGTGACGCCTTCAAAAAGTACGACTTGACTTGTTCTTGTCACATCAGAATCGAAATGAGATCAACTTTTGTAACATGGAGGGAGACGTCTGTGCTTGAATTGCTTAATCATCTCTTGGATTATTGTCCCGACGTCAGTGCGTGTGACACATGATTGTTCAAGAGGGGACTCAAGTATGGAAGTACTTGATCTCTAGGATAATCTACTCCTCCTCGTCGGAGGAACGTACTTACTTTATTATTTTGACTCCCGCCTGATGTGTGTACCGAAGGGCTTTTGTACTGCAAATTACAAATTACCTTAAATAAGTGACCTTCGTAGGCAGTAGGCACCCGCACCTTGCGGGCGTTGCATTGACCCTGTTCATTTACTAAAAGTGAAGTATTTACGAGGTTACTCCGTTCAGAGGGAAATAGGAGGACGAAAAAGTAATTGTGTTCCATTGTTCATACTGCATTCTGATCTGATCTTTTTGTATAGATTGTCAAAATATAGTATTCCAATCAGGCAGCTACTTTTGGCTTATTGCAAATTTGTTTGGGAACATTGAAATTCTAGTTTTTCCAAATTTGTGACTCGATTGAGTCGTAATTTTTTTTACAAATATAGGTAGATTTTTATTTTCACAACTGTCTCCACTTCTTGCTCCCTCGTGTACTTGGACCACTTTGAGCAAAAGTTTCCTCCTTTTCCTTTACTTTTGTGGTTTCGAGATGGTATAGGCTCGTAACTACATACGTTGCTGATGAGTGAAGTGGTGACCTTCCGAGAATAATTGAGGTATAGAAATTGAACGAGTGCTGACATTCACTTGAAAAGTGTGTATTCAAGCATTGTTCACGTTTCCTATTCTTTGATACAAGTTATACACATAGTTTCAAAACCGAAGGCCCTTCTTCGCCCATATTCACCAACCCAGCAGCATCATCTCGTCCCCGTACTGCATTGTATTCCATATTGTATTGTACAAGAGGAGAGTACATCATTTCATACAACATGAACCATAGTGAACAGCCTTTGTCTCTTTGTGCCGACTCATCTCGTCATAAACAAGAAATCTGCTGCTGCTCCTGGCTATGCATCCACGTCCAACTGACACCTTTTACCCGCAGTTTTATAAATTCTCTGAAAGGCTTCCTCAAAGTCAAGATCACTAAATATTCAGCGTTGATTTGTTACAAGTCGAGAGCGAGAGAGATGCGAGTAGTAAAGGATGGATGATGAGGTACAAAATTTCCAGTGAGTCAGTCAGTCAATTGCGGTTTCGTATTGTTGCGTAGAACTGAATGTGTCAAATCAAGATGAAGATAAAGAAAATGAGAAACAACAAGTCGACAAGGGAGCAGCAAAATACGTGGCGTGCATCATGATGCATAAGATCTTCCATTCTGGAGGAGAGTGGAAAAATTTTGGAAAATTGGACACAAAATTATGAATATTTATTTTGAAATATTTGAAATTATGATTTATTAAAAATCGTAGCAGCAACAAATCAAACACTGTTCAAAACTAAAATCTTGTGCAAATCCCATAATTGTTTTGTTGGGAATACTTTTTAAACTCGGCGAGGCTTATGAACCATTCTTCTCCTGTGTATCTGTGCATACGAAGTTTTTTAAGCCGACACCACAAATGGAACTCTTGCACAAAAGTAAAGTGATTTCATTTCGGTGCTTTGCTTTGCTTTACTTTTTTGTCGTTTTATTGACTTCGTGATGACTACAGTGGAGGGATGAATGGCGGTTGTTGGTGTTGTTGTGGACAGGAATTGAATGGTGGTGTAGTGACTCGGACACAAAACCAAGTGGAAAAAATGAATGAGTGCTAATTACAGGAAAAAGTGGAGCTTTTATTTGTCTGCTTTTTAAATGTGGTTGGTGGCATTGTGTGCTCTCTGCCACTGCCAAATTCAGCGCATCATATTTCATTGATGTTTTCCAATTTTAAATATTACTTTGAGTTCAAGTTTATAAGAAGCAATTATGAAATTACGGGATTATCAGAGATATGGTGAATTGAATTTGTGTCATTTTGATGCAAATCTCATGCCAAATAAGTTAAGACCGTGAGTAATAAACGAGATTGCTTAAATAGGCGATTGACCAGATATTTTGCAATGATTAGCACTAACACGCCGTACGAGTCATATGCAATATAAACTGAAACTTCAATTTATTTAGACAAGTATTGAGAAAAAATTCACACTTAAGCCCCACGGATTTGCATCACATGCATCCTTTTAACCCTAGGATGACCATTTTTATGTACATAAGTTATTTTAGCATTTTTTATAAAGTTGAATTTTTTTTCCCTCTGAAAGAACACAACAAGTGTTTACAAACGTGGATCTCTTATTTCTCTCGAGGCTTTTGGGAAAGGAGTGTCCCAATCCTGTCCCCGGAAGCTTCTTCGTCTTCACAACCGCCAGGGATTTCCTCGTCCTTTTCATTTCTCTCAAAATTTCCCCCAAACCCCAAAAAAATCTTATGAAACGGTAGAAGATCTACGATTAAAAATACTACGGACACCCAAACACCATAAATTTTACACTGCTATTTACTTAAATGGAACAGTATTTTTGCGTCTAGCGTGTGCATTTATTTCTGCTTAATTAGCAATCTCTGACTGGAACATTTCCACGTGTTACACAACCTCAACCCTAGGTAGACGTAGGAGTATTCGGGCGTCTCAAATAGCTCAGAGGTACATGATTTTCCAACAATACGACCTCGTATTTACAATACCGCAACCCTAATTTAATTGAACACTGTGTAAAAACACTTTTAGCTCGTACGGCACACGCTGTACAGCACCAAGCATCATTATCGTCATAAAGTTATTATATAAGTGAGTAAAAAGGAGTACGTAAACAAAGTAAACCGTGAAGATTACCAAAGAATTTTTCTCATCAAACTAATGTTTTTCAGCGAAATGAAATATATAATGGTGCACTTTTCTGTTGTACAAAGTCAAATTTTATGCTACACTTGCTTGCATTTACTCATTTTAGGTAGTGGTAAGCAAGGGAGGAATGTGCAGCATGCTGTAGTAAAATCGCATCCATTCATTCCTCCATTCCACTACTTGTTGGAGTACACTTAATTGTTTAATTGTTGAAGGTATTGTAGGAGATTTAGAGATACATATTCAAGATGCGAGATGAGTTATTTCAGACGTAAAGGAATCTGACAACGATATGTATGTGCATATGTGCATATGAAAAGTGTGGAAGAACCTGCACGATGACTTCTCATTTATTACATGCTCCGTGTAACGAGTAATTTCTATTTTCCGTACTATTATTATTATGGATGTAAGCTAAACTATCACTAATTTATTGCATAGGTACAACGCGATAGTGTTGTTCCTAATGCAAAATTACAATGTAGAATGGTCATAAATTTAGAGTCAAAATTCACTTCATGCTCGTTCCTCATGCTCGTACGTACCTACGTAGGTTCCTTATAAATTCGCGTGTGTGTGAATGGGTTTTCTTTTCACAGAGGCACATGAGTTATCGATTTCTGCAAAATTTTTTAGTGGATCAGATAACGCATAAATGGTGTTATCGTCACACTACGCCAAAACTGCTACCAGCTGGTGTACGTGTGCACATACGAGTTTATTCCATCCTGCATAATTCTACTCGCTGACCAATTGACCATTATTAAGTACTATTTTGCATTTTGGGTTGTTTCTTGGCAAGCTATTTGAGGTAAAAGTCCTTGTCAACGTGGTCGTCTTGTGGTGGTTGGTGTGCAGTGGTGGGTTCCATGCCTTGGTGCGTGTGGTTGTGGGCGAAGGCTGTCGTTTACGCTATCCGTGACATGTGGCCAGATTGGTTATGCGGTTTTCGCAATTTGTGGAATCGTGGCAAAAGAACAACAACAATGCGAAACGAGATGCAGTGCAATATCGATCACTTAAAATAAAAAAGTTGTGTGGGTGACATTGTTTTTTATTATCTTGTATTTTTTCTTCTTTCATGCGCACGCACAAGGTTAATATGTAAAATCATAGATATGAAGGGAATTTAGACGAAAACTGCATGCATGCACAAACGTGATGGTTGGTAAATATCTTAAAAAAGTTGTGCTCAGTTTATGATGATCAGACACAAGCTGAAATAATACGAAATGAAGGGAAATGAAACTTTTTAGAGCTAAGACTACATCCCTTCCTTCGCTCGCTTCGAATACGGCCAGTCTGTCAGTAACCAACTGTTTTATCTGGTCTGGCCTTCTTGTCAATCATCATCTCTTACTTTGCTTACCACATGCATTACCTGTTCTCTCGTGTTTTGTGCTATACTAAAATGCAGACTTTTCTATATGATATTCTCACTAAAGCTGCAACATACTTCGTTCCTCGCTGGCTTACATTCACATCACATCACAACATGGCATAATATGATGATGGCAGTAACAAAGGCTGCCAAGCTGCTTCATTTCATTTTATTACTTGTACAATTCCACACCACGTAAATGCATGCATGTAAGTAAGCACCTACTTTTACTTATTTACCACTCGTAAAATAATGCCGATTGGGTGCAGGTACCCACATTTTCGAGTGGCCCTAACAGCCCCATGGATCGAGTAAAGAACACAATTTTGGACATTATTGATTTTGCATGTACACAAATAAATAGGTTCGTTCGTGTTGCAAAATTGAGCTATTTATAATGTAATGTATCTTGTGTCGGATAATTAAAAATGAGATTACCTTTAAAGTAAACATGTAAATAGGGTTGATGAAAAAAGTCATATCCCTCACGGTCCTTGTGTCAGGTTGGTCGGCCCACATTAATTCAATCCCACATTATCCTCGCATCATAGAAAATGAGAGATATCTATGAGTGGAAAAGAATGGAAATATGTGAGTACTACGCGTTTTGTTTGATATGCATGTAGAATAGGTGCGATGTGTGATGGATGGATGGAAGAAAGAGGAGTGTGTAGAGATCATATAAAAGTTTTTAAGCAGATACAAGTACGTATTTGATGCATGTTATGCTCGGACCGGCCCAAACAGCAACCAGCTTGAGATTTTGGTTTGAAATTTTGAGATCAATCTGACAGCTTTTGATGTGGGTTGTCCCAACTCGAGTTAGTTTTCAGGCCATGGGAGATGCATCTCGTCATGATGATCATCAAATCATACCACCTCGCTCGTAAATACAAAGGCCAAACACGTACTCCTTGGATGGATGGAATCCTTCTCGAAGGTTGCTCCATCCCTGCAACACAAAATTTAAATGAATTTCAAGCATTTAGATTAAAATAAAATAATTAATTTCATCAAGAGTATCGTGAAGTTGCGCAATACGTAACTCACTCAGCTCCATTCTACTAAGGAGTATGTACAACTTGATTAAGAATTGCTTACATGATTCGATTAAATTGGAGAAAATGGAGTTGAGCAAATTTAATGTATCAACTGCTCATTATTGTGTAAAATAGTTGACCCAAGAACAGCATTAGATCAGCCATGAATAGGGTAGTACCACCACCAGACAACCAAATATAACAGCTCATTAGACGTCAATGTAGCAGAAAATCTCAGCCGGCTTATTTACCAAGTTACAAATGAATCTCACAAACAAATCCGTAAATCTTGTTGAGCTCTTTCTTCCTTTACCGGTACTTATCCTTAGCCAGAGTAAAGGGACCTGAGGGCCAGACTGGGCGAAAAAGCTTCTTCTTCATCTCGTAACAAGGGCCGACAGCAGCAGGCCAAAAGTTATACAACCTTTATTTTTATTACCAAACCAAACCAAATGAACCGACGGTCGACCACCGCTGCATACCTACCAGCACCTTTATCTACAATGCGATAATACTGCATGTAACAACTAACAAACCGCTAATTGATTTAAATGAATCTTATTCTTAAAGACTTGCTGAACTCGTCGATCTCATCATTCCATTGTTTGCGCAAAAGTTACATAAATACAATTGTAAGATGGAAAAATGATTAAAACAACAAAGTCAAAAGTTGTAAAGATTATCGGAGCACGTATCAATTCATAACCTGACTAATCAACATTTTCACCTTAAATGAGCTATACAGGAAGCTGAGGAGTTGGTTTAGCTGGGGGCACTTTTTGGTCATATGTTCATACTTTTGTGCAAATGACTTTATTCTCCAGTCCTGAAAAAGAAGAGCGTATTCGTTCGTTAGTTAGTTGGTCATCAGGAAGCCTTGCATTATTTTATTACGACCCGCATCATCTCTTTTTTAATAAATTAGTCGTACTTTGTTAGCTGCGTGGCGTGTGTGTGTGGATTATGGTAGTGGCAATAAATTATGGAAAAGCTTAGAAAAATTATTTCCAAAGAACTTCCTCTCTTTATTCCGAAGACTTCCTCAGGCAAATTTTTTCTCGCAGTTCGAAATGCGGAGGAAAACATTATTTTTAGATTAGCATCTCAACTCGCTGTCCCATACGCTTACCTGCCTTCGTATGTGTACCCTTTCCTTGAGAGTAAGGCCGAGTGGGCGTGGGAGGCGTTGTGCTGCAAGTTGGTCGATGTATTTAGATGGCAAAGTTCTCCGTAGGGAAGGTAAGGGTTGCTTTTTACACGGCAGAAGAGTCTCCTCGCATTGTCGGAAGAATAACACTTTGTAGCAATAATATACATAATGTGGACGTAATGCACGGTCAACGGTCCGCCGAACGAGAGTCTCCTCTTAATGATGATCATGTGAACGTACGTAAACATGTACCACGGGTCGGATTTTTTGTGAATTTAAGGTAAAAGTAAAAATATCGTAATCAGGGAAGAGAAATCTGAACGCATGGAATAAAAAATTAGGGTTAAGGGTTAAGATTTCGTGTGGTGGTGTGCGTCTGCCTACATGATCCTGCCTAATGTGGTGGTATGGC

Red:

PFE

Gray:

sequence confirmed by PCR

5’ Genomic region

Putative

Exon1

Putative

Exon2

Putative

Exon3

Putative

Exon4

>FCD Dll full length

ATGCAACACCAGGGGTCCTCGTATAGAGCGGCCGCGGCTGCTGCGGCCGTTGCTGCTTATCAAGAGCAGAACTACAGGTCAGGTGGATATCCCTTCCCCCCACAAAACCCGTACGGGTATCATCTCGGAAACTACCCGCCTCAATGTTCCTCCCCTCCGAAAGATGAGCCGAAAGAGGAGCCGGGTGGAATGCGGTTGAACGGAAAAGGGAAAAAGATGAGAAAACCACGGACAATTTATTCCTCCCTACAATTACAGCAGCTCAACCGCCGCTTCCAGCGCACGCAGTATTTAGCTCTACCTGAACGCGCGGAACTCGCTGCCTCTCTTGGTCTCACACAGACACAGGTGAAAATTTGGTTTCAAAATAGGCGGAGCAAGTACAAGAAGTTGATGAAGGCAGCTCAAGTGCCCGGAGGGTCGAATCAACCGTCGAATCAGCAGGGAGAAAATTCGAATGAGACGATGAGTCCTCAGCCCCCAGATTCTTTCCCTTCGCAACCCGGTGACCTCAGCCCGCCTCCAAATCCTAACCCCCAGTCTCAGGGAGGTTGTTCATCGCCCGTGTCGCCGTGGGATATAAAGGGTGGCGGCGGGGGCGGGGGTCCTCAGCATCAAAACGGTCCCCCCAACATTCCTCAACTTCCCCACCACCCACACCCCGGAATCCCCGTAGGGCACCAACCTTATCAATATCACTGGTACCATCAGGACCACTCCCTTCTCACGTAACTAACACACCTCAACTCTAA
